# MicroRNA‐128 suppresses tau phosphorylation and reduces amyloid‐beta accumulation by inhibiting the expression of GSK3β, APPBP2, and mTOR in Alzheimer's disease

**DOI:** 10.1111/cns.14143

**Published:** 2023-03-07

**Authors:** Siwen Li, Chi Him Poon, Zhigang Zhang, Ming Yue, Ruijun Chen, Yalun Zhang, Md. Farhad Hossain, Yining Pan, Jun Zhao, Lei Rong, Leung Wing Chu, Yat Fung Shea, Ekaterina Rogaeva, Jie Tu, Peter St George‐Hyslop, Lee Wei Lim, You‐Qiang Song

**Affiliations:** ^1^ School of Biomedical Sciences The University of Hong Kong Hong Kong China; ^2^ The Brain Cognition and Brain Disease Institute (BCBDI), CAS Key Laboratory of Brain Connectome and Manipulation, Faculty of Pharmaceutical Sciences, Shenzhen Institute of Advanced Technology, Chinese Academy of Sciences Shenzhen‐Hong Kong Institute of Brain Science‐Shenzhen Fundamental Research Institutions Shenzhen China; ^3^ Tanz Centre for Research in Neurodegenerative Diseases University of Toronto Toronto Ontario Canada; ^4^ Department of Clinical Immunology Third Hospital of Sun Yat‐sen University Guangzhou China; ^5^ Department of Medicine The University of Hongkong‐Shenzhen Hospital Shenzhen China; ^6^ Department of Medicine, LKS Faculty of Medicine The University of Hong Kong, Queen Mary Hospital Hong Kong China; ^7^ The State Key Laboratory of Brain and Cognitive Sciences The University of Hong Kong Hong Kong China

**Keywords:** Alzheimer's disease, amyloid‐beta accumulation, C/EBPα, miR‐128, tau phosphorylation

## Abstract

**Introduction and Aims:**

Alzheimer's disease (AD) is characterized by the abnormal accumulation of hyperphosphorylated tau proteins and amyloid‐beta (Aβ) peptides. Recent studies have shown that many microRNAs (miRNAs) are dysregulated in AD, and modulation of these miRNAs can influence the development of tau and Aβ pathology. The brain‐specific miRNA miR‐128, encoded by *MIR128‐1* and *MIR128‐2*, is important for brain development and dysregulated in AD. In this study, the role of miR‐128 in tau and Aβ pathology as well as the regulatory mechanism underlying its dysregulation were investigated.

**Methods:**

The effect of miR‐128 on tau phosphorylation and Aβ accumulation was examined in AD cellular models through miR‐128 overexpression and inhibition. The therapeutic potential of miR‐128 in AD mouse model was assessed by comparing phenotypes of 5XFAD mice administered with miR‐128‐expressing AAVs with 5XFAD mice administered with control AAVs. Phenotypes examined included behavior, plaque load, and protein expression. The regulatory factor of miR‐128 transcription was identified through luciferase reporter assay and validated by siRNA knockdown and ChIP analysis.

**Results:**

Both gain‐of‐function and loss‐of‐function studies in AD cellular models reveal that miR‐128 represses tau phosphorylation and Aβ secretion. Subsequent investigations show that miR‐128 directly inhibits the expression of tau phosphorylation kinase GSK3β and Aβ modulators APPBP2 and mTOR. Upregulation of miR‐128 in the hippocampus of 5XFAD mice ameliorates learning and memory impairments, decreases plaque deposition, and enhances autophagic flux. We further demonstrated that C/EBPα transactivates *MIR128‐1* transcription, while both C/EBPα and miR‐128 expression are inhibited by Aβ.

**Conclusion:**

Our findings suggest that miR‐128 suppresses AD pathogenesis, and could be a promising therapeutic target for AD. We also find a possible mechanism underlying the dysregulation of miR‐128 in AD, in which Aβ reduces miR‐128 expression by inhibiting C/EBPα.

## Graphical abstract

MiR‐128 can suppress tau phosphorylation by directly targeting GSK3β, and reduce Aβ levels by repressing Aβ generation and enhancing Aβ clearance through targeting APPBP2 and mTOR, respectively. In turn, Aβ can reduce miR‐128 expression by decreasing C/EBPα.

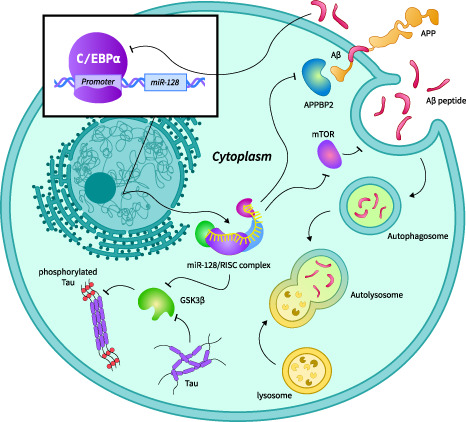

## 1 INTRODUCTION

Alzheimer's disease (AD) is the most common primary cause of dementia in older people and is mainly characterized by memory loss and cognitive impairment. It is becoming one of the greatest global health concerns with increasing incidence worldwide.^1^ There is still no effective therapy to stop or reverse disease progression, which is primarily attributed to the lack of a well‐defined underlying mechanism. Nevertheless, numerous studies have verified the central involvement of amyloid‐beta (Aβ) peptides and tau protein in AD pathogenesis. According to the toxic Aβ oligomers (Aβos) hypothesis,^2^ soluble Aβos, generated from amyloid precursor protein (APP), can affect neuronal membrane integrity and permeability by directly interacting with the membrane or binding to multiple cell surface receptors, induce Ca^2+^ homeostasis dysregulation, mitochondrial damage, reactive oxygen species generation, ATP reduction, and abnormal tau phosphorylation, leading to synaptic dysfunction, neuron loss and impairment of long‐term potentiation. During this process, hyperphosphorylated tau dissociates from microtubules and forms highly insoluble neurofibrillary tangles, disrupting communication and signaling between neurons and eventually leading to neuronal death.^3^ Although tau hyperphosphorylation was initially regarded as a downstream cascade event caused by Aβ aggregation,^4^ evidence shows that tau and Aβ pathologies probably act through parallel pathways to cause AD, while their interaction can also enhance each other's toxicity.

MicroRNAs (miRNAs) are endogenous small noncoding RNAs that repress gene expression through binding to the 3′ untranslated region (UTR) of target messenger RNAs (mRNAs).^5^ Previous studies have demonstrated that miRNAs have pivotal roles in AD, as numerous miRNAs are dysregulated in AD and many miRNAs regulate the expression of genes involved in AD pathogenesis.^6^, ^7^ Increasing evidence shows that restoring or reversing dysregulated miRNAs can counteract AD neuropathology,^8^, ^9^, ^10^, ^11^, ^12^, ^13^ which suggests that the modulation of these dysregulated miRNAs may be a potent therapeutic strategy for AD. There are many advantages when using miRNAs as therapeutic targets for AD. Firstly, a single miRNA can simultaneously regulate several targets, which can modulate disease processes in their entirety and potentially revert a diseased cell to its healthy status. Additionally, the small size of miRNAs facilitates the design of miRNA‐based therapeutic strategies. Moreover, miRNAs can be delivered by multiple established and authorized drug delivery systems such as viral vectors, liposomes, nanoparticles, and exosomes. Therefore, miRNA‐based therapeutics hold much promise as an effective method for future AD treatment.

MicroRNA‐128 (miR‐128), one of the most abundant miRNAs in the adult brain,^14^, ^15^ plays vital roles in neurogenesis and is important for normal brain development.^16^ Several studies have shown that the expression of miR‐128 is dysregulated in AD brains.^17^, ^18^, ^19^, ^20^ However, the role of miR‐128 in AD pathogenesis and the molecular mechanism underlying its dysregulation in AD remain to be elucidated. Here, we found that upregulation of miR‐128 not only suppressed tau phosphorylation and Aβ secretion in AD cellular models but also rescued cognitive impairment and reduced plaque burden in AD transgenic 5XFAD mice. We further demonstrated that miR‐128 exerts its suppressive effects by directly inhibiting the expression of glycogen synthase kinase 3β (GSK3β), amyloid precursor protein binding protein 2 (APPBP2), and the mechanistic target of rapamycin (mTOR). These findings provide the basis for developing novel therapeutics that restore miR‐128 as a treatment for AD. We also discovered that CCAAT/enhancer‐binding protein alpha (C/EBPα) was essential for *MIR128‐1* transcription, and both C/EBPα and miR‐128 expression were found to be repressed by Aβ.

## 2 MATERIALS AND METHODS

### 2.1 Antibodies, reagents, RNA oligoribonucleotides, and vectors

The antibodies and reagents used in this study are listed in Table S1. All miRNA mimics, small interfering RNA duplexes, miR‐128 inhibitor (anti‐miR‐128) and its control (anti‐NC) were purchased from GenePharma (Shanghai, China). The negative control RNA duplex (NC) was nonhomologous to any human genome sequence. The expression vectors, firefly luciferase reporter plasmids of 3′ UTRs, and miR‐128 promoter reporters were constructed as described in the Materials and Methods S1. All constructs were verified by sequencing. The sequences of all RNA oligoribonucleotides and primers are listed in Table S2.

### 2.2 Human brain specimens and animal models

Human cortex tissues from postmortem brains of AD patients and nondemented control subjects were obtained from the Canadian Brain Tissue Bank. The study of human brain tissue was approved by the Ethics Committee of the Faculty of Medicine of the University of Toronto (No: 00026798) and the University of Hong Kong (No: UW 13–177). TgCRND8 mice encoding a double mutant form of APP 695 (KM670/671NL + V717F) and 5XFAD mice coexpressing a total of five AD‐linked mutations [APP K670N/M671L (Swedish) + I716V (Florida) + V717I (London) and PSEN1 M146L + L286V] were used as the AD mouse models. All mice were from a C57BL/6 genetic background. Mice were housed in a temperature‐controlled room with a 12‐h light and dark cycle (lights out at 7 pm) and free access to food and water. All experimental procedures involving animals were approved by the Committee on the Use of Live Animals in Teaching & Research of The University of Hong Kong (No: 4896–18).

### 2.3 Cell culture

Mouse neuroblast Neuro‐2a (N2a) cells, human neuroblastoma SH‐SY5Y cells, and transformed human embryonic kidney (HEK‐293T) cells were maintained in Dulbecco's Modified Eagle's Medium (Gibco, Waltham, MA, USA) supplemented with 10% fetal bovine serum (FBS; Gibco) and 100 units/mL penicillin–streptomycin (Thermo Fisher Scientific, Waltham, MA, USA). The N2a‐APPsw, N2a‐tfLC3, and 293T‐Tau cell lines that were stably transfected with the human Swedish mutant APP695, GFP‐RFP‐LC3, and Tau‐flag, respectively, were constructed as described in the Materials and Methods S1.

### 2.4 Analysis of gene expression

Gene expression was analyzed by real‐time quantitative PCR (RT‐qPCR), Western blotting, or immunofluorescence as described in the Materials and Methods S1.

### 2.5 Enzyme‐linked immunosorbent assay (ELISA)

The N2a‐APPsw cells were transfected with 50 nM NC/miR‐128 duplex or 200 nM anti‐NC/anti‐miR‐128. At 24 h post‐transfection, the medium was discarded and replaced with fresh serum‐reduced medium containing 0.2% FBS. After 24 h of incubation, the cell culture medium was collected and subjected to ELISA assay using the Human β Amyloid (1–42/40) ELISA Kit (Wako, Osaka, Japan) following the manufacturer's instructions.

### 2.6 Luciferase reporter assay

To validate miR‐128‐targeted 3′ UTR, HEK‐293 T cells in a 48‐well plate were cotransfected with 10 nM of NC or miR‐128 duplex, 10 ng of pRL‐TK, and 50 ng of firefly luciferase reporter plasmid that contained either the wild‐type or mutant 3′ UTR of the target gene. To dissect the promoter region of miR‐128, N2a cells grown in a 48‐well plate were cotransfected with 100 ng of firefly luciferase reporter plasmid and 2 ng of pRL‐CMV. To study the effect of C/EBPα and POU2F1 on miR‐128 promoter activity, N2a cells in a 48‐well plate were cotransfected with 2 ng of pRL‐CMV, 100 ng of firefly luciferase reporter plasmid, and 200 ng of gab (empty vector), gab‐C/EBPα (C/EBPα‐expressing vector), or gab‐POU2F1 (POU2F1‐expressing vector). At 48 h post‐transfection, cell lysates were subjected to luciferase assay using the dual‐luciferase reporter system (Promega, Madison, Wisconsin, USA). The pRL‐CMV and pRL‐TK vectors expressing Renilla luciferase were used as the internal controls to adjust for differences in the transfection and harvest efficiencies.

### 2.7 Transmission electron microscopy

The N2a‐APPsw cells were transfected with 50 nM NC or miR‐128 duplex for 48 h, followed by serum starvation for 4 h. Cells were digested by 0.125% trypsin–EDTA and collected in Eppendorf tubes. Cells were then fixed in 2.5% glutaraldehyde in cacodylate buffer (0.1 M sodium cacodylate‐HCl buffer, pH 7.4) for 2 h at 4°C, followed by postfixation in 1% osmium tetroxide (OsO4) in cacodylate buffer for 2 h at 4°C. After fixation, cell pellets were embedded into 2% agarose and the resultant agarose‐embedded cell blocks were processed to dehydration in graded ethanol, followed by infiltration and embedding in pure epon, and finally subjected to polymerization for 72 h in an oven at 60°C. Semithin sections (0.5 μM) were cut and stained with 0.5% toluidine blue to verify the cells were well fixed and embedded. Ultra‐thin sections (80 nM) were cut and sequentially stained with lead citrate and 2% aqueous uranyl acetate. Images were then captured under a Phillips CM100 Transmission Electron Microscope.

### 2.8 Chromatin immunoprecipitation assay (ChIP)

Formaldehyde cross‐linked chromatin complexes were immunoprecipitated using an antibody against C/EBPα or without an antibody (as the negative control) and collected by incubation with Protein L—agarose (Santa Cruz Biotechnology, Dallas, Texas, USA). The DNA‐protein cross‐links were then reversed by heating. The DNA was purified and subjected to PCR and RT‐qPCR using primers covering the C/EBPα binding site on the miR‐128 promoter. Primers used for PCR are listed in Table S2.

### 2.9 Preparation of Aβ42 oligomer (AβO)

The AβO was prepared as described previously.^21^ Briefly, 1 mg of Aβ42 peptide was thawed on ice and dissolved in 221.7 μL of cold HFIP (1, 1, 1, 3, 3, 3‐hexafluor‐o‐2‐propanol; Sigma‐Aldrich, St. Louis, MO, USA). After incubation at room temperature for 1 h to allow Aβ monomerization, the solution was evaporated under a fume hood at room temperature overnight. The film was resuspended by adding 20 μL of dimethyl sulfoxide and 980 μL of F12 medium to obtain a 100 μM stock solution. After incubating at 4°C overnight, the supernatant containing the AβO was collected by centrifugation and stored in aliquots at −80°C.

### 2.10 Primary neuron culture and AβO treatment

Primary cortical neurons were isolated from C57BL/6J embryonic day‐14.5 mice. The tissues were cut into tiny pieces and digested in Tryple Express Enzyme (Thermo Fisher Scientific) supplemented with DNase (Sigma‐Aldrich) for 15 min at 37°C. Cells were plated on poly‐D‐lysine (Sigma‐Aldrich) coated 24‐well plates at a density of 1.5 × 10^6^ cells/mL in Neurobasal™ medium (Thermo Fisher Scientific) supplemented with B‐27 (Thermo Fisher Scientific) and L‐glutamine (Thermo Fisher Scientific). Three days after seeding, cultured cells were exposed to AβO for 24 h and processed for subsequent assays.

### 2.11 Preparation of recombinant adeno‐associated virus (AAV)

The HEK‐293 T cells grown in a 15‐cm cell culture dish were cotransfected with 10 μg pCMV‐miR‐128 or pCMV, 8 μg pXX6 helper plasmid, and 8 μg AAV9 serotype helper plasmid in 100 μL Polyethylenimine (PEI; Polysciences, Warrington, PA, USA) and 1 mL Opti‐MEM I Reduced Serum Medium (Thermo Fisher Scientific, Waltham, MA, USA). At 3 days post‐transfection, the virus was released by repeated freeze–thaw cycles and purified by CsCl density gradient centrifugation. The detailed protocol is described in the Materials and Methods S1.

### 2.12 Stereotaxic injection

Male 5XFAD mice (3 months old) were initially anesthetized with 3% isoflurane vapor mixed with oxygen until loss of reflex. Mice were mounted on a stereotaxic frame and maintained with 1%–1.5% isoflurane through a nose cone. A midline incision was made to expose the skull. A small burr hole was drilled in the skull for the injection. The virus was injected into the dorsal hippocampus according to the coordinates (AP: −2 mm; ML: ±1.5 mm; DV: −1.5 mm) in the Franklin and Paxinos Mouse Brain Atlas. The viral solution [2 μL; AAV9, 2.08 × 10^13^ viral genomes per mL (vg/mL); AAV9‐miR‐128, 1.78 × 10^13^ vg/mL] was injected per hemisphere at a rate of 400 nL/min through a 30G cannula with Hamilton syringe Pump 11 Elite (Harvard Apparatus, Holliston, MA, USA). The injection cannula was left in place for an additional 10 min to allow diffusion of the fluid. The scalp wound was closed with nylon sutures by interrupted suturing. Mice were removed from the stereotaxic apparatus and placed back into their housing cage after regaining normal activity. Mice were subjected to behavioral testing 3 months postinjection and then sacrificed. Brains were dissected and the right cerebral hemisphere was fixed in 4% paraformaldehyde for histological analysis, and the left cerebral hemisphere was frozen at −80°C for biochemistry assays.

### 2.13 Morris water maze

Mice were placed into a large circular pool with a transparent platform placed 1.5 cm below the opaque water surface in the center of a fixed quadrant (target quadrant). Each mouse underwent four trials per day, and the time taken to find the hidden platform was recorded as the escape latency. If the mouse did not find the platform within 60 s, its escape latency was recorded as 60 s. Either way, the mouse was allowed to stay on the platform for 15 s. The intertrial interval for each mouse was 30 min. From day 1 to day 5, mice were placed in the pool at the same four positions but in a different order each day. One day after the training phase, each mouse was subject to the probe trial, in which the hidden platform was removed and the mouse was placed into the water in the opposite quadrant to the target quadrant. The swimming track of the mouse during the 60‐s probe trial was recorded. A GoPro Hero 4 camera (GoPro, San Mateo, CA, USA) was used to record all the trials and the data were analyzed using the ImageJ software with animal tracking plugins.

### 2.14 Histological staining

Paraffin‐embedded brain sections were deparaffinized and rehydrated and subjected to antigen retrieval in sodium citrate buffer (pH 6.0) at 95°C for 10 min. After blocking, the sections were incubated with the primary antibody against Aβ at 4°C overnight. Biotin‐conjugated secondary antibody was added and incubated at 37°C for 30 min. Brown‐colored signals were generated by using the Vectastain ABC system (Vector Laboratories, Burlingame, CA, USA) and diaminobenzidine substrate (Wako). Finally, the sections were counterstained with hematoxylin and mounted for microscopy. Images were taken using an Olympus BX51 Light Microscope (Olympus, Tokyo, Japan). For tracing the GFP expression in brain tissues, paraffin‐embedded slides were deparaffinized, rehydrated, and mounted with Antifade Mounting Medium containing DAPI (Vector Laboratories) for 10 min, followed by the fluorescence imaging using an Olympus BX53 Fluorescence Microscope (Olympus).

### 2.15 Statistical analysis

All data are shown as mean ± SD. The statistical analyses were performed in GraphPad Prism version 9 (GraphPad Software, San Diego, CA, USA). Shapiro–Wilk test for normality was used to assess data distribution. Student's *t*‐test or analysis of variance (ANOVA) was performed for normally distributed continuous data. The Mann–Whitney test or Kruskal‐Wallis test or Friedman test was used when the data was not normally distributed. The correlation between miR‐128 and C/EBPα was analyzed using Spearman's correlation coefficient. A two‐sided test was applied in all statistical tests. *p* < 0.05 was considered statistically significant.

## 3 RESULTS

### 3.1 MicroRNA‐128 suppresses tau phosphorylation and Aβ secretion

**FIGURE 1:**
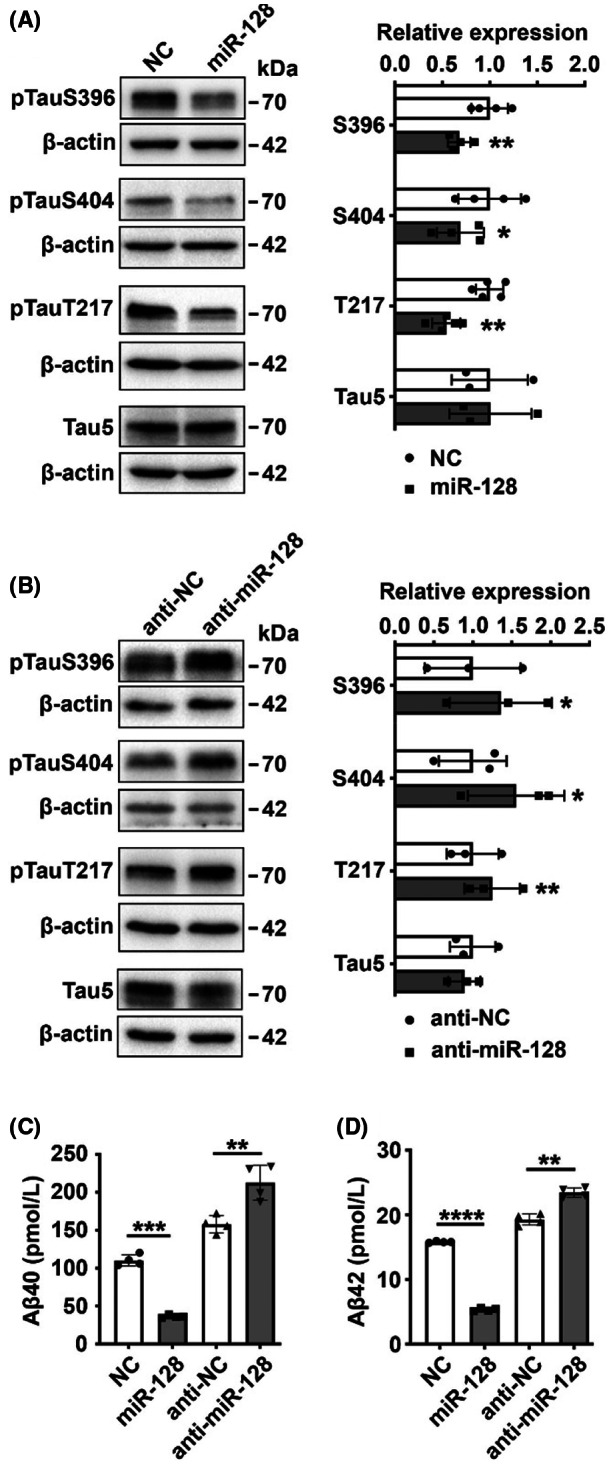
miR‐128 represses tau phosphorylation and reduces secreted Aβ levels. (A, B) miR‐128 suppressed tau phosphorylation at Ser396 (S396), Ser404 (S404), and Thr217 (T217) residues, but did not affect total tau (Tau5) expression. 293T‐Tau cells transfected with negative control (NC) or miR‐128 duplex, miR‐128 inhibitor (anti‐miR‐128), or its control (anti‐NC) for 48 h were analyzed by immunoblotting. β‐Actin was used as a loading control. (C, D) Expression of miR‐128 decreased Aβ40 and Aβ42 secretion, whereas inhibition of miR‐128 increased the secretion. Culture medium from N2a‐APPsw cells transfected with miR‐128 duplex or inhibitor for 48 h were analyzed via ELISA. Data are presented as mean ± SD. *p < 0.05, **p < 0.01, ***p < 0.001, ****p < 0.0001; Student's *t*‐test.

To investigate the role of miR‐128 in AD pathogenesis, we first analyzed the effect of miR‐128 on tau phosphorylation in 293T‐Tau cells that stably express human tau protein. We found that overexpression of miR‐128 repressed tau phosphorylation at serine residues Ser396 and Ser404, and threonine residue Thr217 (Figure 1A), whereas the suppression of miR‐128 enhanced tau phosphorylation at these residues (Figure 1B). In contrast, total tau expression remained unchanged upon miR‐128 overexpression or inhibition (Figure 1A,B). This indicated that the suppressive effects of miR‐128 on tau phosphorylation were not dependent on its effects on total tau, but rather acted through the regulation of kinases or phosphatases. We next evaluated the role of miR‐128 on Aβ accumulation in N2a‐APPsw cells, which is an AD cellular model that generates excessive Aβ peptides by stably overexpressing human APP. ELISA assay revealed that miR‐128 significantly suppressed the secretion of both Aβ40 and Aβ42 (Figure 1C,D), which are the main Aβ peptides involved in the formation of plaques.^22^ These in vitro data suggest that miR‐128 inhibits tau phosphorylation and Aβ secretion.

### 3.2 MicroRNA‐128 represses tau phosphorylation by directly inhibiting GSK3β

**FIGURE 2:**
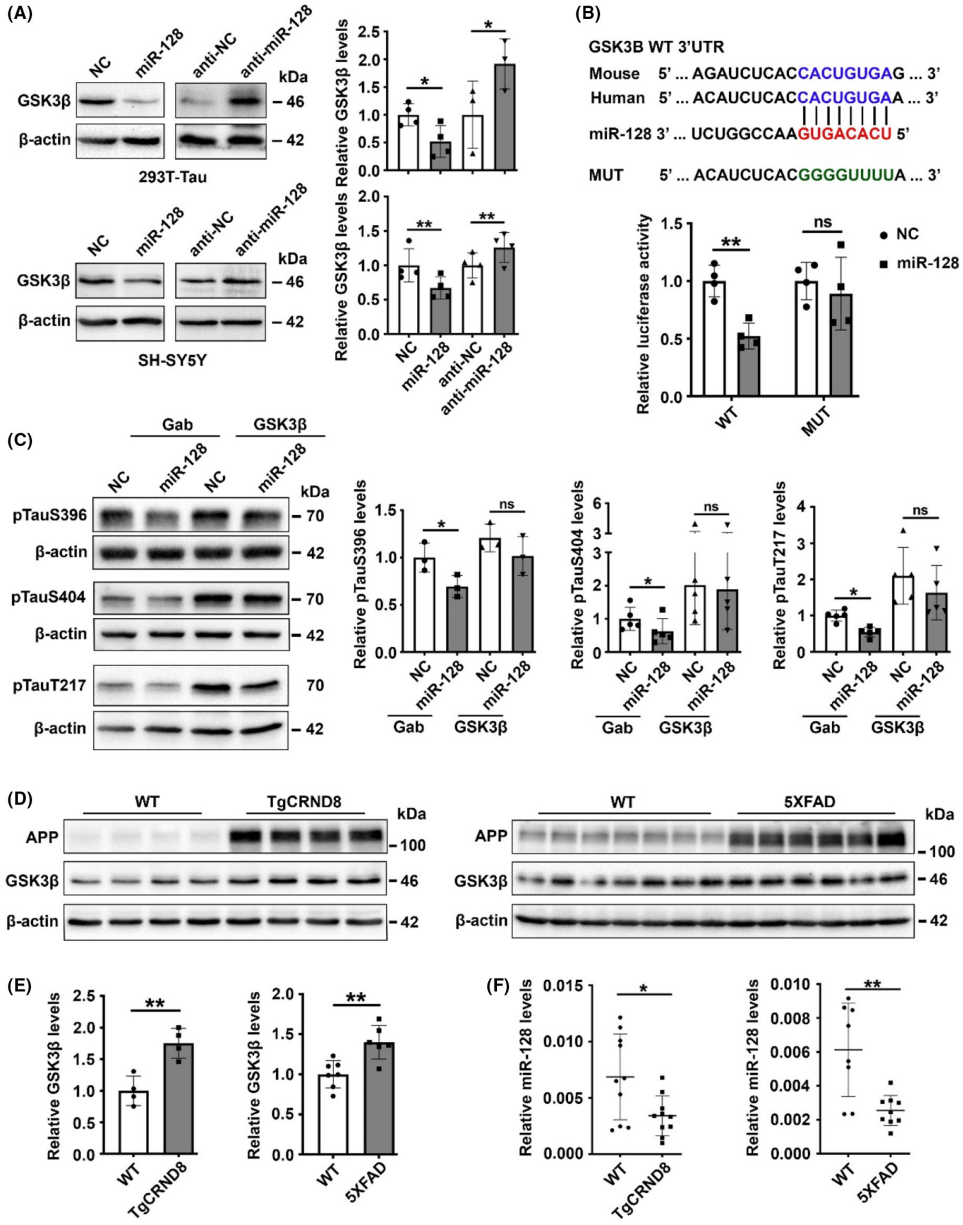
miR‐128 suppresses tau phosphorylation by targeting GSK3β. (A) Expression of miR‐128 reduced cellular GSK3β protein levels. 293T‐Tau cells and SH‐SY5Y cells transfected with miR‐128 duplexes or inhibitors for 48 h were analyzed by immunoblotting. (B) miR‐128 inhibited the activity of the luciferase reporter containing the wild‐type (WT) 3′ UTR of *GSK3B*. NC or miR‐128 duplexes were cotransfected with pRL‐TK and a firefly luciferase reporter plasmid containing either the WT or mutant (MUT) 3′ UTR of *GSK3B*. The pRL‐TK vector expressing Renilla luciferase was used as an internal control to calibrate differences in transfection and harvest efficiencies. The firefly luciferase activity of each sample was normalized to the Renilla luciferase activity. (C) Introduction of GSK3β antagonized the suppressive effects of miR‐128 on tau phosphorylation. 293T‐Tau cells were transfected with NC or miR‐128 duplexes for 24 h. Empty vector (Gab) or GSK3β‐expressing vector (GSK3β) was then transfected into these cells for 48 h according to the following combinations: NC/Gab (first bar), miR‐128/Gab (second bar), NC/GSK3β (third bar), or miR‐128/GSK3β (fourth bar). Phosphorylated tau expression was subsequently assessed. (D) GSK3β expression was upregulated in the hippocampus of AD transgenic mice. Hippocampus of 9‐month‐old WT, TgCRND8, and 5XFAD mice were homogenized and subjected to Western blotting. (E) Comparison of GSK3β levels in WT and AD transgenic mice using data from (D). (F) miR‐128 was downregulated in the hippocampus of AD transgenic mice. The expression level of mature miR‐128 in the hippocampus of 9‐month‐old WT and AD transgenic mice was analyzed by RT‐qPCR. Data are presented as mean ± SD. *p < 0.05, **p < 0.01, ns, nonsignificant; Student's *t*‐test for results in (A, E, F), One‐way ANOVA for results in (B, C).

We next explored the molecular mechanism underlying the inhibitory role of miR‐128 on tau phosphorylation. In silico prediction of miR‐128 targets was performed using the TargetScan tool. Among the multiple candidates, GSK3β was chosen for further validation due to its well‐known function in tau phosphorylation.^23^ An immunoblotting assay demonstrated that overexpression of miR‐128 repressed cellular GSK3β expression, whereas inhibition of miR‐128 upregulated GSK3β expression (Figure 2A). Additionally, the dual‐luciferase reporter analysis revealed that cotransfection with miR‐128 significantly inhibited the activity of the firefly luciferase reporter containing the wild‐type 3′ UTR of GSK3β mRNA, whereas this effect was abolished when the putative binding site was mutated (Figure 2B). We further investigated whether GSK3β mediated the suppressive effect of miR‐128 on tau phosphorylation. We showed that introduction of GSK3β attenuated the inhibitory effects of miR‐128 in 293T‐Tau cells (Figure 2C), whereas suppression of GSK3β reduced the tau phosphorylation induced by miR‐128 inhibition (Figure S1). Moreover, GSK3β was upregulated in the hippocampus of TgCRND8 and 5XFAD mice (Figure 2D,E), in which miR‐128 was downregulated (Figure 2F), suggesting a negative correlation between GSK3β and miR‐128 expression in AD transgenic mice. Taken together, these findings imply that miR‐128 may suppress tau phosphorylation by directly targeting GSK3β.

### 3.3 MicroRNA‐128 reduces Aβ levels by suppressing its generation via inhibiting APPBP2

It has been demonstrated that miR‐128 is a tumor suppressor, which can induce cell apoptosis and inhibit cell proliferation.^24^, ^25^ To exclude the possibility that the decreased Aβ secretion by miR‐128 may be due to reduced cell viability or cell apoptosis, we performed TUNEL and XTT assays to assess cell apoptosis and proliferation in N2a‐APPsw cells under the same conditions as in the previous ELISA analysis. We found that miR‐128 did not induce cell apoptosis or inhibit cell proliferation (Figure S2), suggesting the inhibitory function of miR‐128 on Aβ secretion is independent of these two pathways.

**FIGURE 3:**
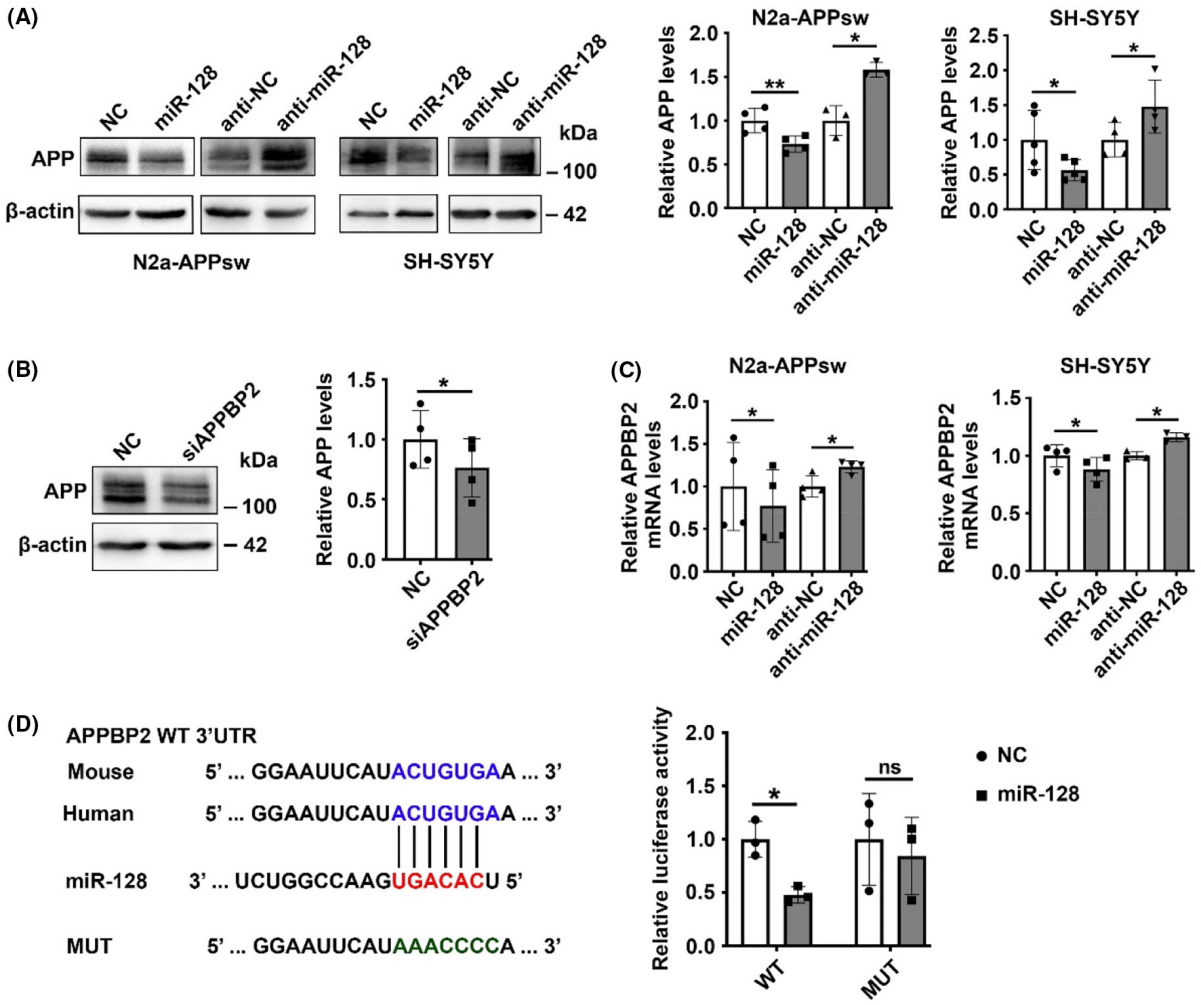
miR‐128 suppresses APP expression by directly inhibiting APPBP2. (A) miR‐128 repressed cellular APP expression. N2a‐APPsw cells and SH‐SY5Y cells transfected with miR‐128 duplexes or inhibitors for 48 h were analyzed by immunoblotting. (B) Knockdown of APPBP2 reduced cellular APP protein levels. N2a‐APPsw cells were transfected with siRNAs targeting APPBP2 (siAPPBP2) for 48 h, followed by immunoblotting analysis. (C) miR‐128 suppressed the mRNA level of APPBP2 in cells transfected with miR‐128 duplexes for 48 h. (D) miR‐128 inhibited the activity of the luciferase reporter containing the wild‐type (WT) 3′ UTR of *APPBP2*. Luciferase activity analysis was performed as in Figure 2B. Data are presented as mean ± SD. *p < 0.05, **p < 0.01, ns, nonsignificant; Student's *t*‐test for results in (A–C), One‐way ANOVA for results in (D).

We next explored the mechanism underlying the suppressive effect of miR‐128. As the abnormal accumulation of Aβ in AD is induced by its increased production or/and decreased clearance, we first tested whether miR‐128 regulates Aβ generation. Among the predicted targets of miR‐128, beta‐site APP cleaving enzyme 1 (BACE1) and presenilin 1 (PSEN1) stood out as candidates of interest, because they participate in the cleavage of APP to generate neurotoxic Aβ peptides.^26^ Our results showed that overexpression of miR‐128 inhibited APP expression (Figure 3A), but did not affect either BACE1 or PSEN1 expression (Figure S3). Although miR‐128 does not directly bind to APP mRNA, we found a possible direct target, APPBP2, an APP binding protein that stabilizes the localization of APP in the cell membrane to facilitate Aβ generation.^27^ Consistently, knockdown of APPBP2 inhibited APP expression (Figure 3B). Meanwhile, upregulation of miR‐128 repressed APPBP2 mRNA levels and suppressed the activity of the firefly luciferase reporter that contained the wild‐type 3′ UTR of APPBP2 mRNA (Figure 3C,D). These results indicate that miR‐128 can inhibit APP expression by directly targeting APPBP2, resulting in reduced Aβ levels.

### 3.4 MicroRNA‐128 decreases Aβ levels by enhancing its clearance via inhibiting mTOR

**FIGURE 4:**
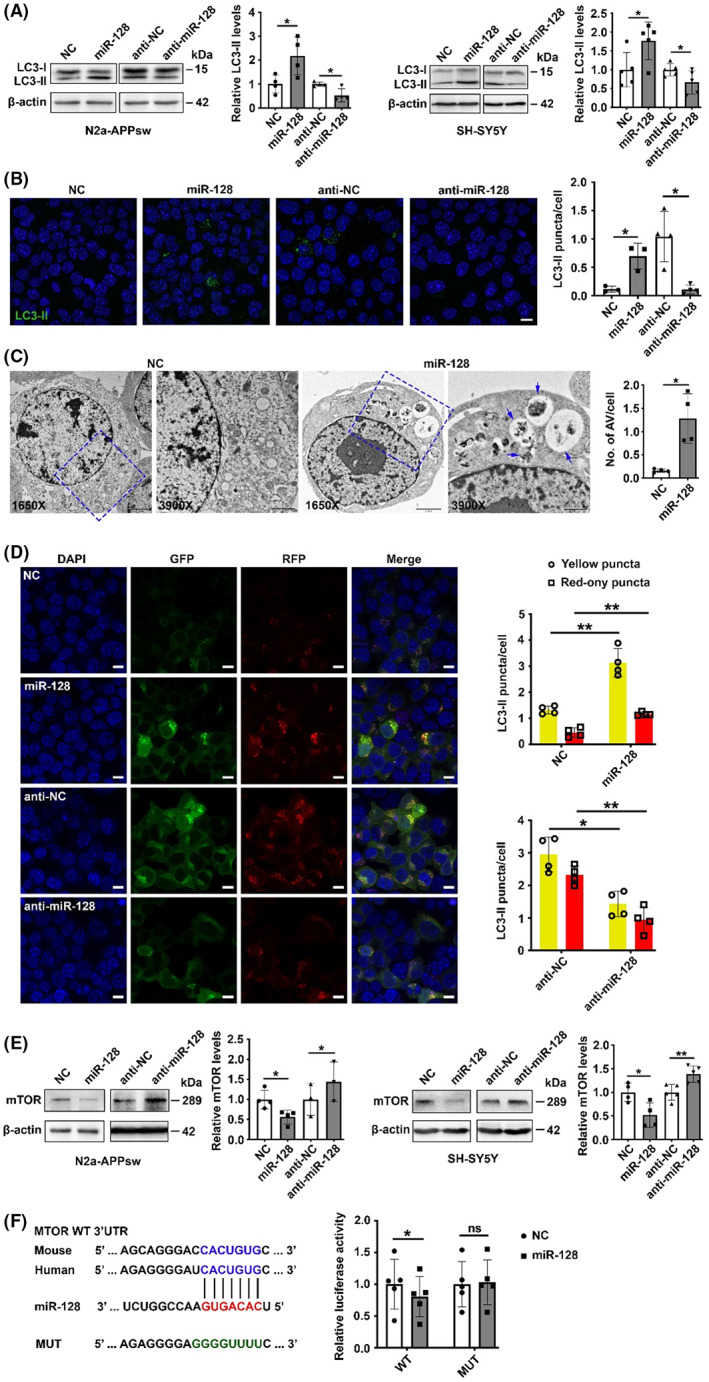
miR‐128 promotes autophagy by directly inhibiting mTOR. (A) miR‐128 increased cellular LC3‐II expression. N2a‐APPsw cells and SH‐SY5Y cells transfected with miR‐128 duplexes or inhibitors for 48 h were treated with serum starvation for 2 or 4 h, respectively, followed by immunoblotting. (B) miR‐128 increased LC3‐II positive puncta. An immunofluorescence assay was used to analyze the LC3‐II positive puncta in N2a‐APPsw cells transfected with miR‐128 duplexes or antagonists. Cells transfected with antagonists were subject to serum starvation for 2 h before testing. Scale bar, 10 μm. (C) miR‐128 increased the number of autophagic vacuoles. N2a‐APPsw cells transfected with NC or miR‐128 for 48 h were subject to serum starvation for 4 h before transmission electron microscopy. Arrows indicated AVs. Scale bar, 2 μm in 1650×, 1 μm in 3900×. AV, autophagic vacuole. (d) miR‐128 enhanced autophagic flux. N2a‐tfLC3 cells were transfected with miR‐128 duplexes or inhibitors for 48 h, and cells transfected with inhibitors were treated with serum starvation for 2 h before the immunofluorescence assay. Yellow puncta represent autophagosomes and red‐only puncta represent autolysosomes. Scale bar, 10 μm. (e) miR‐128 decreased cellular mTOR expression level. (f) miR‐128 inhibited the activity of the luciferase reporter containing the WT 3′ UTR of *MTOR*. Luciferase activity analysis was performed as in Figure 2B. Data are presented as mean ± SD. *p < 0.05, **p < 0.01, ns, nonsignificant; Student's *t*‐test for results in (A–E), One‐way ANOVA for results in (F).

We next investigated whether miR‐128 can regulate the evolutionarily conserved autophagy clearing process,^28^ whose impairment induces cognitive deficits.^29^, ^30^, ^31^ To this end, we analyzed the expression of LC3‐phosphatidylethanolamine conjugate (LC3‐II), a characteristic marker of the autophagosome,^32^ in cells transfected with miR‐128 duplexes or inhibitors. We found that overexpression of miR‐128 increased LC3‐II protein levels, whereas inhibition of miR‐128 decreased LC3‐II expression (Figure 4A,B). This result suggests that miR‐128 promotes the formation of autophagosomes, which was further validated by transmission electron microscopy (Figure 4C).

To exclude the possibility that miR‐128 might increase LC3‐II expression by impeding the fusion of autophagosomes with lysosomes, we constructed an N2a‐tfLC3 stable cell line, in which LC3 is coexpressed with both green fluorescent protein (GFP) and red fluorescent protein (RFP) so that LC3‐II positive autophagosomes are present with yellow fluorescence. However, when the pH is lower than 7, such as in the acidic environment of the autolysosome, the green fluorescence will be quenched, and only the red fluorescence is left to trace LC3 expression. Therefore, yellow puncta and red‐only puncta represent autophagosomes and autolysosomes in the autophagy process, respectively. Our results revealed that overexpression of miR‐128 increased the number of both yellow puncta and red‐only puncta, whereas inhibition of miR‐128 decreased both puncta (Figure 4D). We also found that miR‐128 overexpression decreased the expression of p62, an autophagy cargo receptor, while miR‐128 suppression increased the expression (Figure S4a). Additionally, treatment with Bafilomycin A1, an inhibitor of autophagosome‐lysosome fusion, enhanced the increase of LC3‐II level induced by miR‐128 overexpression (Figure S4b). These results indicate that miR‐128 can enhance autophagic flux.

We next explored the mechanism by which miR‐128 promotes autophagy. We investigated mTOR because it is an important negative regulator of autophagy and has been experimentally validated as a direct target of miR‐128 in glioma cells.^33^ The immunoblotting assay and dual‐luciferase reporter analysis verified that miR‐128 suppressed the expression of mTOR by directly targeting its 3′ UTR (Figure 4E,F).

In summary, our results suggest that miR‐128 can promote autophagic flux by directly targeting mTOR, resulting in enhanced Aβ clearance and decreased Aβ levels.

### 3.5 Administration of miR‐128 ameliorates cognitive deficits and reduces plaque burden in 5XFAD mice

**FIGURE 5:**
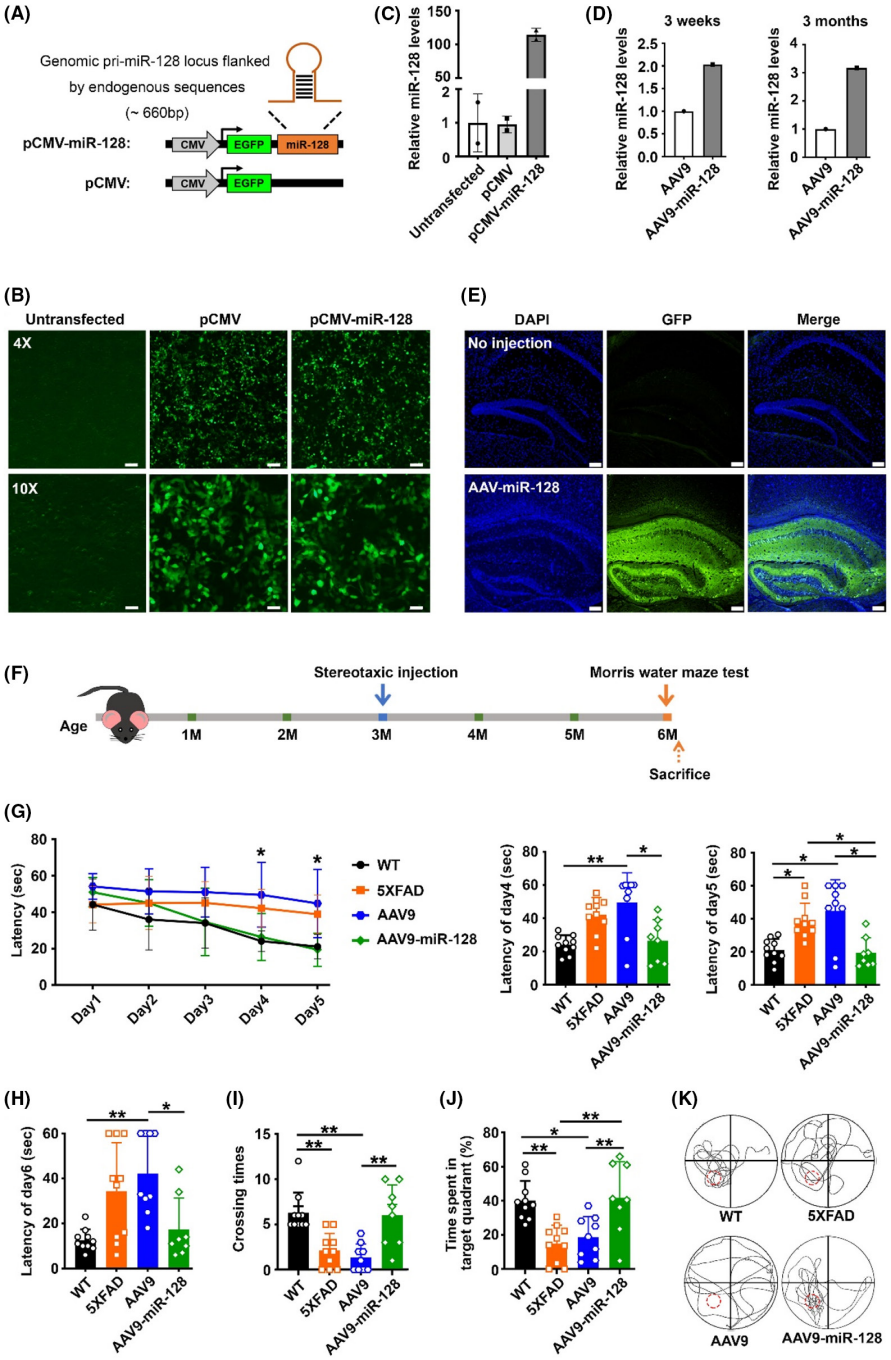
Administration of miR‐128 rescues cognitive impairment in 5XFAD mice. (A) Schematic representation of the AAV plasmid construct encoding the pri‐miR‐128 sequence. The primary sequence of miR‐128 was cloned into an AAV backbone plasmid (pCMV) harboring enhanced green fluorescent protein (EGFP) reporter gene driven by a human cytomegalovirus (CMV) promoter. (B) Fluorescence microscopy showing equivalent GFP expression in HEK‐293T cells transiently transfected with the indicated AAV vectors. Scale bar, 115 μm in 4×; 47 μm in 10×. (C) RT‐qPCR demonstrating a significant upregulation of miR‐128 in HEK‐293T cells transiently transfected with miR‐128‐expressing vector. (D) RT‐qPCR showing expression of miR‐128 in hippocampus 3 weeks and 3 months following administration of the indicated AAVs. (E) Fluorescence microscopy displaying widespread GFP expression throughout the hippocampus of 5XFAD mice 3 months postadministration of AAV9‐miR‐128. Scale bar, 100 μm. (D–E) 5XFAD mice at 3 months were stereotaxically injected with 2 μL of AAV9‐miR‐128 into the right hippocampal dentate gyrus (DG) region and 2 μL of AAV9 into the left hippocampal DG region. Brain tissues were harvested at 3 weeks and 3 months postinjection for analyzing miR‐128 and GFP expression. (F) Schematic representation of the experimental time course. 5XFAD male mice at 3 months were stereotaxically injected in the hippocampus of both hemispheres with AAVs. Cognitive function was assessed by Morris water maze 3 months postinjection. (G) Escape latency to the hidden platform in the Morris water maze was measured during the consecutive 5‐day training trial. (H–K) Latency to reach the previously hidden platform area (H), crossing time (I), time spent in the target quadrant (J), and the representative swimming path (K) in the probe trial of the Morris water maze were analyzed. (G–K) WT, wild‐type mice, *n* = 10; 5XFAD, untreated 5XFAD mice, *n* = 10; AAV9, 5XFAD mice injected with control virus, *n* = 9; AAV9‐miR‐128, 5XFAD mice injected with miR‐128‐expressing virus, *n* = 8. (K) The red line circle indicated the previous location of the hidden platform. Data are presented as mean ± SD. *p < 0.05, **p < 0.01; Kruskal‐Wallis test for results in (G–I), One‐way ANOVA for results in (J).

To evaluate the therapeutic potential of miR‐128 in 5XFAD mice, we first constructed a recombinant adeno‐associated virus (AAV) vector system, in which miR‐128 is coexpressed with an enhanced green fluorescent protein (EGFP) (Figure 5A). We tested if this vector efficiently expressed both GFP and miR‐128 by transient transfection of HEK‐293T cells. We found an equivalent GFP expression between cells transfected with miR‐128‐expressing vector pCMV‐miR‐128 and the control vector pCMV (Figure 5B). We also observed a dramatic increase in miR‐128 expression in pCMV‐miR‐128‐transfected cells (Figure 5C). We next packaged pCMV‐miR‐128 and pCMV with the AAV9 vector to generate viral particles (referred to as AAV9‐miR‐128 and AAV9, respectively) for in vivo delivery. To assess the efficiency and stability of the viral vectors for neuronal infection, we performed stereotaxic injection of AAVs into the hippocampal dentate gyrus (DG) region of 5XFAD mice. We found the expression of miR‐128 at either 3 weeks or 3 months postinjection was higher in the AAV9‐miR‐128‐injected group than in the AAV9‐injected group (Figure 5D). Fluorescence microscopy of brain sections also displayed widespread GFP expression throughout the hippocampus in mice injected with viral particles (Figure 5E).

**FIGURE 6:**
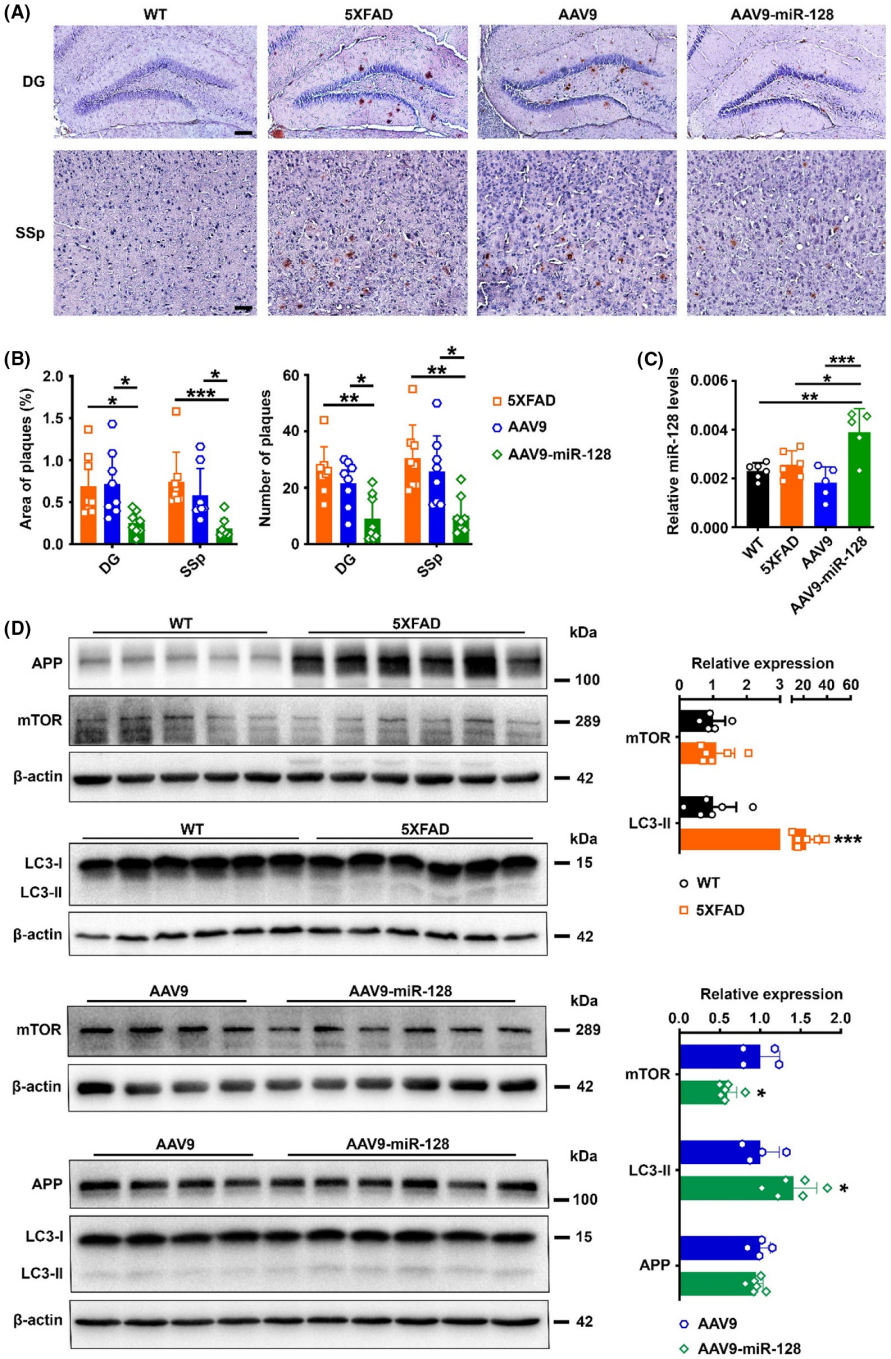
AAV9‐mediated miR‐128 expression reduces plaque burden, decreases mTOR, and increases LC3‐II expression in 5XFAD mice. (A, B) Representative microscopy images (A) and statistical results (B) of plaque burden in experimental mice. DG, dentate gyrus; SSp, primary somatosensory cortex. Scale bar, 100 μm in DG, 25 μm in SSp. *n* = 8 for each group. (C) RT‐qPCR analysis revealed a significant increase in miR‐128 expression in the hippocampus of AAV9‐miR‐128‐injected 5XFAD mice. Wild‐type (WT) and 5XFAD, *n* = 6; AAV9 and AAV9‐miR‐128, *n* = 5. (d) Expression of APP, mTOR, and LC3‐II in the hippocampus of experimental mice. WT, *n* = 5–6; 5XFAD, *n* = 6; AAV9, *n* = 4; AAV9‐miR‐128, *n* = 6. Data are presented as mean ± SD. *p < 0.05, **p < 0.01, ***p < 0.001; One‐way ANOVA for results in Area of plaques in DG and Number of plaques in SSp from (b) and results in (c), Kruskal‐Wallis test for results in Area of plaques in SSp and Number of plaques in DG from (b), Mann–Whitney test for results in mTOR expression between AAV9 and AAV9‐miR‐128 from (d), Student's *t*‐test for other results in (d).

To explore the long‐term therapeutic efficacy of the delivery of miR‐128 for the treatment of AD, 5XFAD mice were bilaterally injected with AAV9‐miR‐128 or AAV9 in the hippocampal DG region. The virus was administered at 3 months of age, at which point the mice have amyloid plaque deposits but show normal cognitive performance (Figure S5), which should mimic therapeutic administration at the early stage of AD. At 3 months postinjection, the mice were subjected to the Morris water maze test to evaluate learning and memory (Figure 5F). Compared with uninfected and AAV9‐infected 5XFAD mice, AAV9‐miR‐128‐infected 5XFAD mice had shorter latency from day 4 of the training trial (Figure 5G), and shorter latency to first crossing, more crossings to the platform region, and longer swimming time in the target quadrant during the probe trial (Figure 5H–K). This suggests a significant improvement in spatial learning and memory in 5XFAD mice treated with AAV9‐miR‐128. In addition, immunohistochemical staining for Aβ peptides in brain sections showed reduced plaque burden in the hippocampus and cortex of AAV9‐miR‐128‐infected 5XFAD mice (Figure 6A,B). These data indicate the administration of miR‐128 in the hippocampus of 5XFAD mice rescued cognitive impairment and reduced plaque deposition.

### 3.6 In vivo upregulation of miR‐128 decreases mTOR and p62 while increases LC3‐II expression

We next investigated whether the therapeutic effects of miR‐128 are due to the regulation of target genes as shown in our previous in vitro results. We first measured the expression of miR‐128 in the hippocampus of experimental mice by RT‐qPCR, which showed significantly upregulated miR‐128 levels in AAV9‐miR‐128‐infected 5XFAD mice (Figure 6C). We then analyzed the hippocampal expression of APP, mTOR, p62, and LC3‐II by Western blotting, which showed mTOR and p62 were not altered between wild‐type mice and uninfected 5XFAD mice but were decreased in AAV9‐miR‐128‐infected mice compared to AAV9‐infected mice (Figure 6D, Figure S8). In contrast, LC3‐II was significantly upregulated in 5XFAD mice and was further upregulated in AAV9‐miR‐128‐infected mice (Figure 6D). There was no significant difference in APP expression between AAV9‐infected and AAV9‐miR‐128‐infected mice (Figure 6D), which might be due to the overwhelming expression of APP in 5XFAD mice.

### 3.7 MicroRNA‐128 is transactivated by C/EBPα

Our data strongly suggest that miR‐128 suppresses AD pathogenesis, and its downregulation might contribute to AD progression. Therefore, understanding the mechanism underlying the reduction of miR‐128 in AD could facilitate future drug development. MicroRNA‐128 is a type of intronic miRNA encoded by two distinct genes, *MIR128‐1* and *MIR128‐2*, which are embedded in the introns of *R3HDM1* and *ARPP21*, respectively (Figure S6a). Both *MIR128‐1* and *MIR128‐2* can be processed to generate identical mature miR‐128 with the same sequence. It is commonly recognized that the majority of intronic miRNA transcripts are dependent on their host gene promoters and can be processed from the same primary transcript. We thus analyzed the hippocampal transcripts of miR‐128 host genes in mice, which showed no differences in R3HDM1 and ARPP21 mRNA levels between wild‐type and AD transgenic mice (Figure S6b), indicating a poor correlation between the transcription of miR‐128 and its host genes in AD transgenic mice. One feasible explanation is that in addition to the host gene promoter, miR‐128 may have its transcriptional regulatory elements for expression independent of the intron. Indeed, it has been predicted that 35% of intronic miRNAs may have their intronic promoters that can drive transcription independently of the host gene promoters.^34^ Both *MIR128‐1* and *MIR128‐2* have been predicted to have putative intronic promoter activity within their 5′ flanking regions.^34^

**FIGURE 7:**
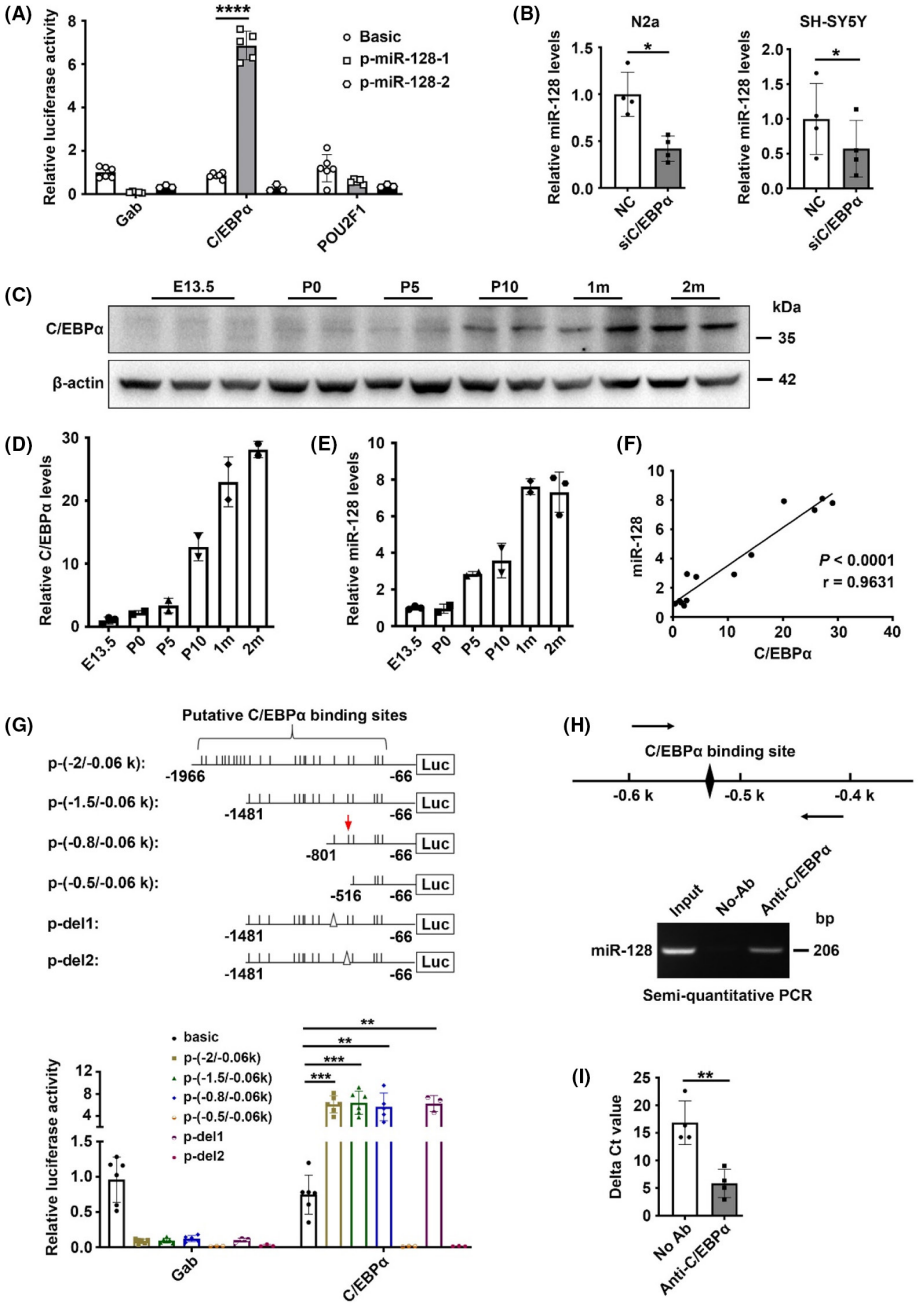
C/EBPα is essential for *MIR128‐1* transcription. (A) Ectopic expression of C/EBPα increased the luciferase activity of p‐miR‐128‐1. N2a cells were cotransfected with pGL4‐basic (Basic), or p‐miR‐128‐1 or p‐miR‐128‐2, and pRL‐CMV, and either control (Gab) or C/EBPα‐expressing (C/EBPα) or POU2F1‐expressing (POU2F1) vector for 48 h before luciferase activity analysis. (B) Knockdown of C/EBPα reduced endogenous miR‐128 level. The expression level of mature miR‐128 was analyzed by RT‐qPCR in N2a and SH‐SY5Y cells transfected with NC or siC/EBPα for 48 h. (C–E) Expression of C/EBPα and miR‐128 were upregulated during brain development. Cortex tissues were collected from fetuses (E13.5d), postnatal (0, 5, 10 d, 1 m; birth day as day 0), and adult (2 months) wild‐type mice and homogenized to analyze the expression levels of C/EBPα (C, D) and miR‐128 (E). (F) A positive correlation was observed between C/EBPα and miR‐128 during brain development. Correlation was determined using Spearman's correlation coefficient. (G) The 5′ deletion and site‐specific deletion analysis revealed that the C/EBPα binding site located in the −0.6 to −0.5 kb region is essential for C/EBPα to transactivate *MIR128‐1* transcription. Short vertical lines represent putative C/EBPα binding sites and the triangle (∆) depicts deletion of the C/EBPα binding site. (H) ChIP assay showed that C/EBPα directly interacted with *MIR128‐1* in vivo. The scheme of the amplicons is shown at the top. The C/EBPα binding site located in the −0.6 to −0.5 kb region is depicted as a black diamond and primers used for the PCR are represented as arrows. Anti‐C/EBPα, C/EBPα antibody‐precipitated DNA group. No‐Ab, no antibody‐precipitated DNA group. (I) qPCR analysis showed enrichment of the miR‐128 promoter sequence in the Anti‐C/EBPα‐precipitated DNA compared to no antibody‐precipitated DNA using DNA from (H). The Ct value of each group was subtracted from the input group to give the ∆Ct value. Data are presented as mean ± SD. *p < 0.05, **p < 0.01, ***p < 0.001, ****p < 0.0001; Student's *t*‐test for results in (A, B, I), One‐way ANOVA for results in (G).

We thus cloned the 2 kb genomic sequence upstream of mature miR‐128 to a promoterless vector to generate the p‐miR‐128‐1 and p‐miR‐128‐2 reporter constructs (Figure S6c). We observed no significant increase in luciferase activity in N2a cells transfected with the two reporters (Figure S6c), which is in agreement with the extremely low endogenous miR‐128 level in the cells (Figure S6d). This may imply that the transcription of miR‐128 in N2a cells is maintained at a very low level, primarily due to the low levels of transcription factors (TFs) that are essential for miR‐128 transcription. To identify potential TFs, we analyzed several putative binding sites of TFs within the 2 kb region upstream of miR‐128, which revealed the top four candidates listed in Table S3. The top two candidates, C/EBPα and POU2F1, were selected for further validation, as they are also involved in AD according to genetic studies (Table S3). Luciferase reporter assay showed the overexpression of C/EBPα significantly increased the activity of p‐miR‐128‐1, but not p‐miR‐128‐2, whereas overexpression of POU2F1 did not affect the activity of these two reporters (Figure 7A). Meanwhile, knockdown of C/EBPα significantly reduced endogenous miR‐128 expression (Figure 7B). Moreover, the expression pattern of C/EBPα was similar to that of miR‐128 during brain development (Figure 7C–E), and both expressions were significantly positively correlated (Figure 7F). These results suggest that C/EBPα can transactivate *MIR128‐1* transcription.

Twenty‐five C/EBPα consensus binding sites were predicted within the −2 to −0.06 kb region upstream of *MIR128‐1*. To validate the functional binding site (s), we performed a 5′ deletion analysis (Figure 7G). Compared with the activity of the p‐(−2/−0.06 k), there was no significant change in either p‐(−1.5/−0.06 k) or p‐(−0.8/−0.06 k) (Figure 7G). However, a dramatic decrease was observed in the activity of p‐(−0.5/−0.06 k) (Figure 7G), implying that the region between −0.8 and − 0.5 kb is essential for C/EBPα to transactivate *MIR128‐1* transcription. Within this region, two C/EBPα consensus binding sites were predicted (denoted as site 1 and site 2, respectively; Figure 7G). Deletion of site 2 (p‐del2), but not site 1 (p‐del1), significantly attenuated p‐(−1.5/−0.06 k) activity (Figure 7G). Moreover, ChIP analysis revealed an interaction between C/EBPα and site 2 in vivo (Figure 7H,I). These data indicate that C/EBPα binding site 2 located in the −0.8 to −0.5 kb region is crucial for C/EBPα to transactivate *MIR128‐1* transcription.

### 3.8 C/EBPα and miR‐128 are inhibited by Aβ

**FIGURE 8:**
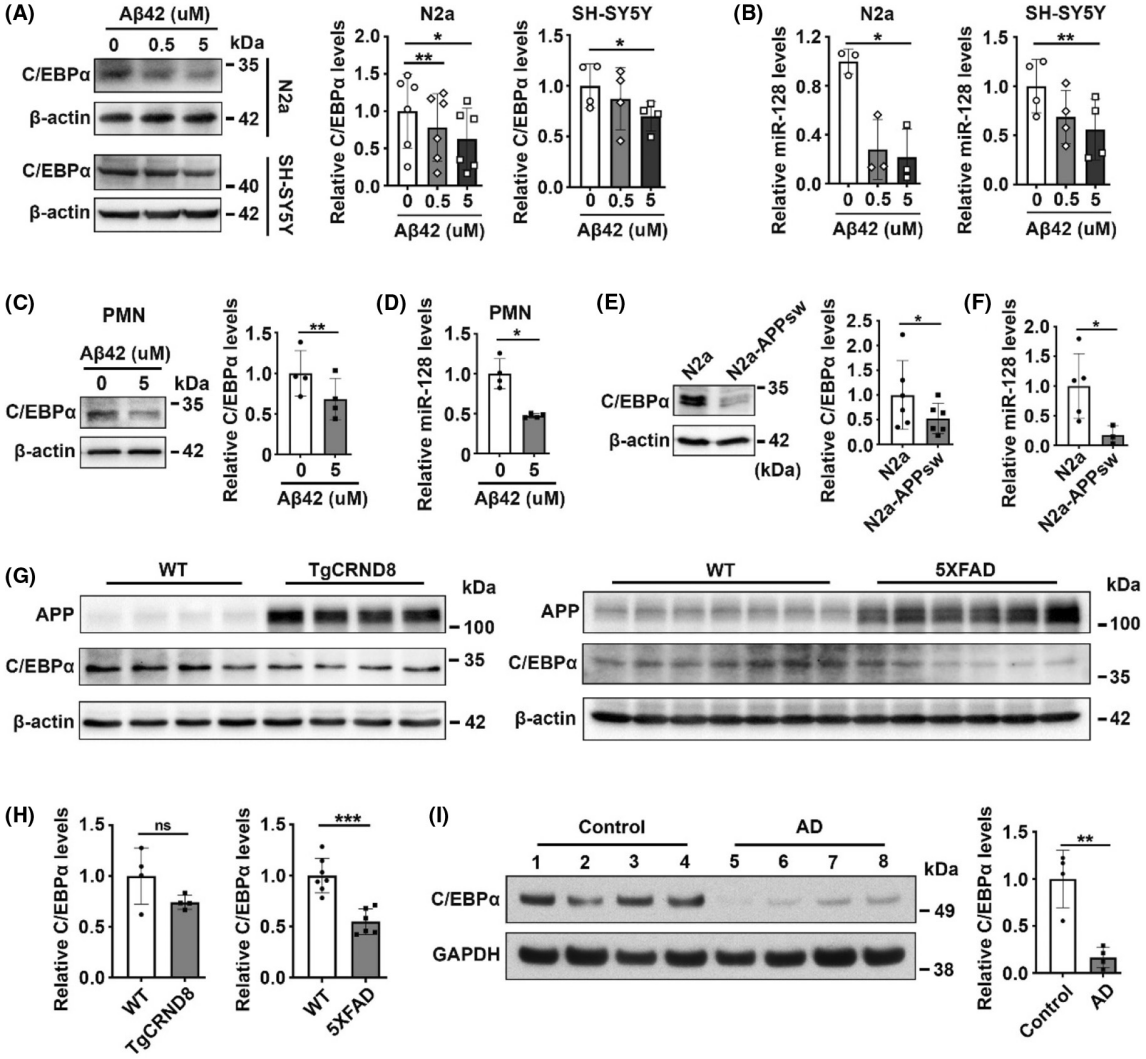
Aβ represses C/EBPα and miR‐128 expression. (A–D) Treatment with Aβ42 oligomers suppressed C/EBPα (A, C) and miR‐128 (B, D) expression in N2a and SH‐SY5Y cells as well as primary mouse neurons. Cells were treated with Aβ42 oligomers for 24 h, followed by immunoblotting and RT‐qPCR analysis. PMN, primary mouse neurons. (E, F) Expression of C/EBPα (E) and miR‐128 (F) in N2a and N2a‐APPsw cells. (G) C/EBPα was downregulated in the hippocampus of TgCRND8 and 5XFAD mice. Hippocampus of 9‐month‐old wild‐type (WT), TgCRND8, and 5XFAD mice were homogenized and processed for Western blotting to analyze C/EBPα expression. (H) Comparison of the C/EBPα expression levels in the hippocampus of WT and AD transgenic mice using data from (G). (I) C/EBPα was decreased in AD postmortem brain. C/EBPα expression was assessed in frontal cortical brain lysates from normal control and AD tissues by immunoblotting. Data are presented as mean ± SD. *p < 0.05, **p < 0.01, ***p < 0.001, ns, nonsignificant; Student's *t*‐test for results in (C–F, H, I), One‐way ANOVA for results in (A) and results in miR‐128 expression levels of SH‐SY5Y cells from (B), Friedman test for results in miR‐128 expression levels of N2a cells from (B).

As it has been reported that treatment with Aβ42 peptides can lead to reduced C/EBPα levels in microglial cells derived from human postmortem brain,^35^ we tested whether Aβ42 peptides could also decrease C/EBPα expression in neuronal cells. As expected, our results showed the Aβ42 oligomer treatments in N2a and SH‐SY5Y cells, as well as primary mouse neurons, reduced both C/EBPα and miR‐128 expression (Figure 8A–D). In addition, the expression levels of C/EBPα and miR‐128 were lower in N2a‐APPsw cells than in N2a cells (Figure 8E,F). Moreover, we also found decreased C/EBPα levels in the hippocampus of TgCRND8 and 5XFAD mice (Figure 8G,H), and the entorhinal cortex of AD postmortem brain (Figure 8I). These findings suggest that Aβ can repress C/EBPα and miR‐128 expression, which might explain the decrease of miR‐128 in AD.

## 4 DISCUSSION

Alzheimer's disease is characterized by the deposition of hyperphosphorylated tau proteins and Aβ peptides, and extensive studies have validated the crucial roles of tau and Aβ in AD pathogenesis. Therefore, miRNAs that possess suppressive effects on tau phosphorylation and Aβ accumulation may provide novel targets for AD treatment. In this study, we found in vitro that miR‐128 not only suppressed tau phosphorylation by inhibiting GSK3β but also reduced Aβ accumulation by repressing its generation and enhancing autophagy through inhibiting APPBP2 and mTOR, respectively. We also showed in vivo that upregulation of miR‐128 in 5XFAD mice alleviated learning and memory impairments, reduced plaque deposition, and enhanced autophagy. Our findings suggest that miR‐128 suppresses AD pathogenesis, and could be a therapeutic target for AD.

It should be noted that other studies have found different effects of miR‐128 in AD. Carrettiero et al.^36^ showed that upregulation of miR‐128 in primary rat neurons resulted in increased tau levels via repressing the ubiquitin/proteasome system by directly inhibiting the expression of Hsc70 cochaperone BAG2. However, we provided evidence that overexpression of miR‐128 in 293 T‐Tau cells did not affect total tau expression, but suppressed tau phosphorylation by inhibiting GSK3β expression (Figures 1A, 2A–C). Consistently, data in primary mouse neurons also revealed that upregulation of miR‐128 inhibited tau phosphorylation and GSK3β expression (Figure S7). In addition, administration of miR‐128 in the hippocampus of 5XFAD mice had no effect in total tau expression but repressed tau phosphorylation at serine residue Ser404, though no difference was observed in GSK3β expression (Figure S8), which suggested that there might be other mechanism mediating the suppressive effect of miR‐128 on tau phosphorylation. This speculation was confirmed by our findings that overexpression of miR‐128 increased phosphorylated (inactive) GSK3β level (Figure S9). These data indicated that miR‐128 suppressed tau phosphorylation not only by inhibiting GSK3β expression but also through repressing its activity. Nevertheless, further research is required to clarify the overall effects of miR‐128 on tau pathology by using an in vivo tau transgenic mouse model, since 5XFAD mouse model mainly recapitulates the major features of amyloid pathology but not tau pathology.^37^

Another study discovered that upregulation of miR‐128 in monocytes repressed the expression of lysosomal enzymes and inhibited monocyte‐mediated Aβ degradation.^38^ A more recent study demonstrated that the knockout of miR‐128 in 3 × Tg‐AD transgenic mice alleviated the AD‐like pathologies by inhibiting PPARγ.^39^ These conflicting findings are not surprising, because one miRNA can bind up to hundreds of target mRNAs due to the short base pairs within the miRNA‐3′ UTR duplex,^40^ resulting in different or even contrary roles mediated by different targets in a specific disease, as seen with other miRNAs such as miR‐132 in tau phosphorylation^9^, ^41^ and miR‐200 in tumor metastasis.^42^, ^43^ Compared to the two studies, our results are more convincible in demonstrating the role of miR‐128 in AD. On one hand, we verified the inhibitory role of miR‐128 on Aβ accumulation in both in vitro and in vivo AD models that are more relevant to AD than monocytes. On the other hand, unlike the total knockout of miR‐128 in the whole genome of 3 × Tg‐AD transgenic mice, we upregulated miR‐128 expression in the hippocampus of 5XFAD mice by stereotaxic injection of miR‐128‐expressing AAVs, which could furthest minimize side effects caused by the whole genome editing. Moreover, miR‐128 is important for brain development, and miR‐128 deficiency can induce hyperactivity and premature death in mice, with all miR‐128‐deficient mice dying before 4 months of age.^44^ Hence, it is impossible that miR‐128‐knockout 3 × Tg‐AD mice could survive for up to 12 months without showing any abnormality.^39^

Our data revealed that miR‐128 was reduced in the hippocampus of TgCRND8 and 5XFAD mice, while this reduction only occurred in the late stage of AD, as we found that miR‐128 expression was not altered between TgCRND8 mice and wild‐type mice at an early age (<8 months) but was significantly decreased in TgCRND8 mice at 8 months or older (Figure S10). Similarly, we observed downregulated miR‐128 in the hippocampus of 9‐month‐old 5XFAD mice (Figure 2F), but not in 6‐month‐old 5XFAD mice (Figure 6C). Together with the findings that Aβ suppressed miR‐128 expression (Figure 8B,D), we infer that miR‐128 is downregulated in the late stage of AD as the concentration of Aβ increases in the AD brain.

Intriguingly, results on miR‐128 expression levels in human AD brains are inconsistent between different studies (Table S4). This inconsistency may be caused by different sample types (e.g., neocortex, hippocampus, etc.), different collection protocols, different measurement methods (microarray, RNA sequencing, and RT‐qPCR), and different sample sizes across studies. Also, considering the relatively short half‐life of miRNAs in tissues, an inconsistent postmortem interval between patient death and brain freezing may result in unreliable miR‐128 expression in the AD brain. In addition, miR‐128 may display a time‐dependent expression profile during the progression of AD,^20^ so studies on different disease status may lead to less sophisticated results. Therefore, standardizing laboratory procedures, employing uniform methods, and conducting studies with relatively larger sample size will be important to overcome these problems and get more reliable and reproducible results in human samples. Although results in human AD brains are controversial at present, the expression level of miR‐128 in blood samples of AD patients seems to be upregulated (Table S4), but large‐scale and multicenter studies are also required for further validation.

Although C/EBPα is a well‐known tumor suppressor in acute myeloid leukemia and other solid tumors,^45^, ^46^, ^47^, ^48^ little is known about its function in AD. A genetic study revealed that AD risk variants and AD heritability were significantly enriched in a subset of open chromatin sites containing DNA binding motifs for specific TFs including C/EBPα.^49^ Additionally, recent studies on the risk of AD identified the significance of microglia, whose differentiation was found to be dependent on C/EBPα.^50^ Herein, we showed that C/EBPα was decreased in Aβ‐treated cells, AD transgenic mice, and AD postmortem brains. We also discovered that C/EBPα was essential for *MIR128‐1* transcription. These findings suggest that restoration of C/EBPα may be a novel potential therapeutic strategy for AD.

In conclusion, we found that miR‐128 suppresses tau phosphorylation by targeting GSK3β and reduces Aβ accumulation by repressing Aβ generation and enhancing Aβ clearance via targeting APPBP2 and mTOR. We also identified a plausible mechanism behind the dysregulation of miR128 in AD, in which Aβ reduces miR‐128 expression by decreasing C/EBPα. It is intriguing to find that a single miRNA can regulate the two important pathological processes in AD. Such a miRNA may represent a promising molecular target for AD therapies.

## AUTHOR CONTRIBUTIONS

SWL designed and performed the experiments, analyzed the data, and drafted the manuscript; CHP performed the stereotaxic injection experiments; ZGZ, MY, RJC, YLZ, and YNP performed the experiments and analyzed the data; MFH and JZ revised the manuscript; LR, LWC, YFS, ER, JT, and PSH guided the research, reviewed the data, and revised the manuscript; LWL and YQS designed and guided the research, reviewed the data, and revised the manuscript.

## CONFLICT OF INTEREST STATEMENT

The authors declare no conflict of interest.

## Supporting information


Appendix S1:

Figure S1:

## Data Availability

All data generated during this study have been included in this manuscript and its supplementary information file. Further data supporting the findings of this study are available from the corresponding author on request.
