# Supplementary material for: MicroRNA‐128 suppresses tau phosphorylation and reduces amyloid‐beta accumulation by inhibiting the expression of GSK3β, APPBP2, and mTOR in Alzheimer's disease
Source: CNS Neurosci Ther. 2023 Mar 7;29(7):1848–64. doi: 10.1111/cns.14143 (PMC10324361; doi:10.1111/cns.14143)
Supplement: Supplementary file 1 — Appendix S1: [file CNS-29-1848-s002.docx]

**Supplementary Information**

**MicroRNA-128 suppresses tau phosphorylation and reduces amyloid-beta accumulation by inhibiting the expression of GSK3β, APPBP2, and mTOR in Alzheimer’s disease**

Siwen Li, Chi Him Poon, Zhigang Zhang, Ming Yue, Ruijun Chen, Yalun Zhang, Md. Farhad Hossain, Yining Pan, Jun Zhao, Lei Rong, Leung Wing Chu, Yat Fung Shea, Ekaterina Rogaeva, Jie Tu, Peter St George-Hyslop, Lee Wei Lim*, and You-Qiang Song*

* Corresponding authors: You-Qiang Song, School of Biomedical Sciences, The University of Hong Kong, Hong Kong, China, [songy@hku.hk](mailto:songy@hku.hk). Phone: +852 3917 9245 Fax: +852 2855 1254 and Lee Wei Lim, [limlw@hku.hk](mailto:limlw@hku.hk).

**Inventory**

**A) Supplementary Materials and Methods pages 2-9**

**B) Supplementary Tables pages 10-14**

**C) Supplementary References pages 15-17**

**Supplementary Materials and Methods**

**Vector construction**

The expression vectors gab-POU2F1 and gab-GSK3β were constructed by cloning the coding sequences of POU2F1 (2301 bp) or GSK3β (1263 bp) into the *KpnI* and *EcoRV* sites of pcDNA3.1+ vector (Addgene, Watertown, MA, USA).

To construct the miR-128-targeted 3′ UTR vectors, wild-type 3′ UTR mRNAs of human *GSK3B* (594 bp), *APPBP2* (505 bp), and *MTOR* (950 bp) containing putative binding sites for miR-128 were inserted into *EcoRI* and *ApaI* sites, *EcoRV* and *XbaI* sites, or *EcoRI* and *EcoRV* sites, respectively, downstream of the stop codon of firefly luciferase in pGL3cm [[1](#_ENREF_1)], which was re-edited based on the pGL3-control vector (Promega, Madison, WI, USA). The resultant vectors were designated as pGL3cm-GSK3B-3′ UTR-WT, pGL3cm-APPBP2-3′ UTR-WT, and pGL3cm-MTOR-3′ UTR-WT, respectively.

To construct pGL3cm-GSK3B-3′ UTR-MUT, pGL3cm-APPBP2-3′ UTR-MUT, and pGL3cm-MTOR-3′ UTR-MUT, the complementary sites of the seed region of miR-128 within the 3′ UTRs were mutated by site-specific mutagenesis using pGL3cm-GSK3B-3′ UTR-WT, pGL3cm-APPBP2-3′ UTR-WT, and pGL3cm-MTOR-3′ UTR-WT as the templates, respectively.

The adeno-associated virus (AAV) expression vector pCMV-miR-128 was constructed by inserting a genomic fragment (665 bp) encompassing the corresponding pri-miR-128 and its 5′- and 3′- flanking sequences into *XhoI* and *EcoRV* sites of the pCMV vector (kindly given by Dr. Yun Cheng). In this expression vector, the coding sequence of miR-128 was fused to the 3′ terminal of the CDS of enhanced green fluorescent protein (EGFP). The expression of miR-128 and EGFP were both driven by a CMV promoter.

To dissect the promoter region of miR-128, the indicated genomic sequences upstream of miR-128 were inserted upstream of the firefly luciferase in the pGL4.20 vector (Addgene). To construct p-del1 or p-del2, the putative C/EBPα binding site 1 (AAATGAATGT), or site 2 (AACAAAATAAA) located between the -0.8 to -0.5 kb region, respectively, was deleted by fusion PCR using p-(-1.5/-0.06 k) as the template.

All constructs were confirmed by direct sequencing. All primer sequences used for cloning are listed in Supplementary Table S4.

**Establishment of stable cell lines**

Stable cell lines N2a-APPsw, N2a-tfLC3, and 293T-Tau were established by virus infection and subsequent drug selection. Briefly, for N2a-APPsw construction, N2a cells were infected with a lentivirus expressing APPsw (human APP695 with Swedish mutation) and puromycin resistance protein. At 48 hours post-infection, cells were subjected to selection in the presence of 2 μg/mL puromycin for 1 week. For N2a-tfLC3 establishment, N2a cells were infected with a lentivirus expressing GFP-RFP-LC3 and neomycin resistance protein. At 48 hours post-infection, cells were subjected to selection with 800 μg/mL G418 for 2 weeks. For constructing a 293T-Tau stable cell line, HEK-293T cells were infected with a lentivirus expressing Tau and puromycin resistance protein. At 48 hours post-infection, cells were subjected to selection with 2 μg/mL puromycin for 1 week. The final stable cell lines were maintained in DMEM supplemented with 10% FBS and 1% penicillin-streptomycin.

**Real-time quantitative PCR (****RT-qPCR)**

Total RNA from cells or tissues was prepared using Trizol reagent (Invitrogen, Waltham, MA, USA). For analyzing the expression of miRNAs, first-strand complementary DNA (cDNA) was synthesized using a TaqMan miRNA reverse transcription kit (Applied Biosystems, Foster, CA, USA). For measuring the expression of other genes, total RNA was reverse transcribed into cDNA using a PrimeScript™ RT Reagent Kit (Takara, Shiga, Japan). The cDNA was used as the template for RT-qPCR using TB Green Premix Ex Taq™ (Tli RNase H Plus, Takara). All RT-qPCR reactions in each experiment were run in triplicate. The expression levels of miR-128 were normalized to that of U6, whereas the expression levels of other genes were normalized to that of GAPDH or β-actin to calculate the 2^-∆Ct^ value. Primers for RT-qPCR are listed in Supplementary Table S4.

**Western blotting**

Protein samples from cells or tissues were prepared in cold RIPA buffer supplemented with protease and phosphatase inhibitors (Roche, Basel, Switzerland). Equal amounts of protein samples were separated in SDS-polyacrylamide gels and then transferred to nitrocellulose membranes (GE Healthcare, Chicago, IL, USA). Membranes were blocked with 5% BSA solution, followed by incubation with primary antibodies overnight at 4°C. After incubation with secondary antibodies at room temperature for 1 hour, the proteins were detected by the ChemiDoc XRS + system (Bio-rad, Hercules, CA, USA) using SuperSignal® West Pico Chemiluminescent Substrate (Thermo Fisher Scientific).

**Terminal deoxynucleotidyl transferase dUTP nick end labeling (TUNEL)**

Coverslips were placed into a 24-well plate and coated with Poly-D-lysine hydrobromide (Sigma Aldrich) at room temperature for 4 hours. After discarding the Poly-D-lysine hydrobromide, the coated coverslips were rinsed three times with PBS and sterilized under UV light for 30 minutes. N2a-APPsw cells were seeded on the coverslips and transfected with 50 nM NC or miR-128 duplex. At 24 hours post-transfection, the medium was discarded and replaced with fresh serum-reduced medium (0.2% FBS). After 24 hours, the medium was discarded and cells were fixed with 4% paraformaldehyde at room temperature for 30 minutes. After washing three times with PBS, cells were subjected to the TUNEL assay using an In Situ Cell Death Detection Kit (Roche). Briefly, cells were incubated in permeabilization solution (0.1% Triton X-100 in 0.1% sodium citrate) at room temperature for 8 minutes, rinsed twice with PBS before adding 50 μL of the TUNEL reaction mixture, and incubated at 37°C in the dark for 60 minutes. For preparing the positive control, cells were incubated with recombinant DNase I at room temperature for 30 minutes to induce DNA strand breaks before incubating with the TUNEL reaction mixture. Finally, cells were embedded with DAPI-containing Antifade Mounting Medium and observed under the Zeiss LSM700 confocal microscope and analyzed with Zeiss ZEN software (Zeiss, Oberkochen, Germany).

**XTT assay for cell proliferation**

The N2a-APPsw cells were seeded onto a 96-well plate at a concentration of 2 × 10^4^ cells per well and transfected with 50 nM NC or miR-128 duplex. At 24 hours post-transfection, the medium was discarded and replaced with fresh serum-reduced medium (containing 0.2% FBS). After 24 hours, cells were subjected to XTT assay by adding 50 μL of the XTT labeling mixture (Cell Proliferation Kit, Roche) and incubated at 37°C and 5% CO2 for 4 hours. The absorbance of the sample was measured at 475 nm using a SpectraMax 340 Plate Reader (Molecular Devices, San Jose, CA, USA).

**Immunofluorescence**

To investigate the role of miR-128 on LC3-II expression, N2a-APPsw cells were seeded on coverslips placed in a 24-well plate and transfected with 50 nM NC/miR-128 duplex or 200 nM anti-NC/anti-miR-128 for 48 hours. For cells transfected with inhibitors, the medium was replaced with fresh medium without FBS 2 hours before fixation. Cells were washed once with PBS and fixed with 4% paraformaldehyde at room temperature for 30 minutes. After washing with PBS, cells were blocked with 5% BSA (in PBST containing 0.1% Triton X-100) at room temperature for 1 hour. Cells were incubated with primary antibody (LC3B, 1: 250 in PBST) overnight at 4°C, followed by the incubation with secondary antibody (Alexa Fluor® 488, 1: 200 in PBST) for 1 hour in the dark at room temperature. Finally, the coverslips were mounted with a drop of Antifade Mounting Medium containing DAPI (Vector Laboratories) and incubated for 10 minutes at room temperature. The fluorescent images were captured under a Zeiss LSM980 confocal microscope and analyzed with Zeiss ZEN software (Zeiss).

To explore the effect of miR-128 on the autophagic flux, N2a-tfLC3 cells were seeded on coverslips placed in a 24-well plate and transfected with 50 nM NC/miR-128 duplex or 200 nM anti-NC/anti-miR-128 for 48 hours. For cells transfected with inhibitors, the medium was replaced with fresh medium without FBS 2 hours before fixation. Cells were washed with PBS and fixed with 4% paraformaldehyde at room temperature for 30 minutes. After fixation, cells were mounted with a drop of Antifade Mounting Medium containing DAPI and incubated at room temperature in the dark for 10 minutes. The fluorescent images were captured under a Zeiss LSM700 confocal microscope and analyzed with Zeiss ZEN software (Zeiss).

**Preparation of recombinant adeno-associated virus (AAV)**

*Cell transfection*

The HEK-293T cells were seeded in a 15-cm dish and cotransfected with 10 μg of the AAV expression vector (pCMV-miR-128 or pCMV), 8 μg of pXX6 helper plasmid, and 8 μg of AAV9 serotype helper plasmid in 100 μL of Polyethylenimine (PEI, 1 mg/mL) and 1 mL Opti-MEMTM I Reduced Serum Medium (Thermo Fisher Scientific).

*Virus purification*

At 3 days post-transfection, cells were harvested by scraping and then centrifuged at room temperature at 1000 rpm for 10 minutes. The cell pellets were washed twice with PBS and re-suspended with 6 mL PBS, followed by six repeated freeze-thaw cycles in liquid nitrogen and in a 37°C water bath to release the virus. Next, benzonase (Sigma Aldrich) was added and incubated at 37°C for 30 minutes, followed by the addition of deoxycholate and incubation at room temperature for a further 30 minutes. After centrifugation, the viral supernatant was filtered through a 0.45-μm membrane and placed in the upper layer of a 10-mL ultra-clear centrifuge tube layered with 3 mL of 1.3 g/cm^3^ CsCl in the middle and 3 mL of 1.5 g/cm^3^ CsCl in the bottom. After centrifugation at 17°C, 60,000 rpm for 1 hour, the viral solution residing at the interface between the 1.3 and 1.5 g/cm^3^ CsCl solutions was transferred into a new 10-mL ultra-clear centrifuge tube layered with 1.4 g/cm^3^ CsCl in the bottom, and centrifuged at 50,000 rpm overnight at 17°C. After a second CsCl gradient centrifugation, the supernatant was divided into 10 x 1 mL fractions (named 10 to 1 counting from the bottom of the tube). Six to 10 fractions containing high viral copy number were collected and dialyzed with AAV dialysis/storage solution using Millipore Centricon® to give ~200 μL vector stock, which was stored in aliquots at -80°C.

*Virus titration*

The virus titer of the samples was measured according to the protocol described on the Addgene website. Briefly, 2 μL of purified virus samples were added to 50 μL of DNase I digestion buffer and incubated at 37°C for 1 hour to eliminate any contaminating plasmid DNA, 2.5 μL of 0.5 M EDTA was added and incubated at 70°C for 10 minutes to inactive DNase I, 8 μL of Proteinase K was added and incubated at 50°C for 4 hours to release the virion DNA, followed by a final 10-min incubation at 100°C to inactive Proteinase K. After six serial dilutions with standard plasmid (pCMV-miR-128, 2 × 10^9^ molecules/μL), the final dilution concentrations ranged from 2 × 10^3^ to 2 × 10^8^ molecules/μL. Likewise, the DNase-treated AAV samples were subject to serial dilution with concentrations ranging from 1000× to 625,000×. The diluted standard solutions and AAV samples were subject to qPCR analysis. The titer of the samples was calculated based on the standard curve and the sample dilution factors.

**Supplementary Tables**

**Table S1. Antibodies and reagents**

| **Antibodies** | **Vendor & Catalog #** |
| --- | --- |
| p-Tau S396 | Abcam, ab109390 |
| p-Tau S404 | Sigma-Aldrich, T7444 |
| p-Tau T217 | Thermo Fisher Scientific, 44744 |
| Tau5 | Thermo Fisher Scientific, AHB0042 |
| GSK3β | BD Biosciences, 610201 |
| pGSK3β | Cell Signaling Technology, 9323 |
| APP (N-terminus) | Millipore, MAB348 |
| BACE1 | Sigma-Aldrich, SAB2100200 |
| Presenilin 1 (C-terminus) | Abcam, ab15456 |
| β-actin | Sigma-Aldrich, A5316 |
| GAPDH | Abcam, ab8245 |
| LC3 | Novus Biologicals, NB100-2220 |
| mTOR | Cell Signaling Technology, 2972 |
| p62 | Santa Cruz Biotechnology, sc-28359 |
| C/EBPα | Santa Cruz Biotechnology, sc-365318 |
| β-amyloid, 1-16 (6E10) | Biolegend, 803001 |
| Goat anti-Rabbit IgG (H+L) Secondary Antibody, HRP | Thermo Fisher Scientific, 31460 |
| Goat anti-Mouse Secondary Antibody, HRP | Bio-Rad, 170-6516 |
| Goat anti-Mouse IgG (H+L) Secondary Antibody, Biotin | Boster Biological Technology, BA1001 |
| Goat anti-Rabbit IgG (H+L) (Alexa Fluor ® 488) | Abcam, ab150077 |
|  |  |
| **Reagents & Kits** | **Vendor & Catalog #** |
| Cell Proliferation Kit (XTT) | Roche, 11465015001 |
| Human β Amyloid(1-42) ELISA Kit | Wako, 298-62401 |
| Human β Amyloid(1-40) ELISA Kit | Wako, 292-62301 |
| Dual-Luciferase® Reporter Assay System | Promega, E1960 |
| In Situ Cell Death Detection Kit, TMR Red | Roche, 15129800 |
| TaqMan™ MicroRNA Reverse Transcription Kit | Thermo Fisher Scientific, 4366597 |
| PrimeScript™ RT Reagent Kit with gDNA Eraser (Perfect Real Time) | Takara, RR047A |
| TB Green® Premix Ex Taq™ (Tli Rnase H Plus), ROX Plus | Takara, RR42WR |
| Polyethylenimine (PEI) | Polysciences, 23966 |
| Benzonase Nuclease | Sigma Aldrich, E1014 |
| Cesium Chloride (CsCl) | Carl Roth, 7878.5 |
| Antifade Mounting Medium with DAPI | Vector Laboratories, H-1200 |
| Beta-Amyloid (1-42) | rPeptide, A-1002-2 |
| B-27 Serum-Free Supplement | Life Technologies, 17504-044 |
| L-Glutamine | Thermo Fisher Scientific, A2916801 |

**Table S2. Sequences of RNA and DNA oligonucleotides**

| **Name** | **Sense Strand/Sense Primer (5’-3’)** | **Antisense Strand/Antisense Primer (5’-3’)** | |
| --- | --- | --- | --- |
| **miRNA and siRNA duplexes** | | | |
| hsa/mmu-miR-128-3p | UCACAGUGAACCGGUCUCUUU | AGAGACCGGUUCACUGUGAUU | |
| si-mmu-C/EBPα | GCCGCUGGUGAUCAAACAAdTdT | UUGUUUGAUCACCAGCGGCdCdG | |
| si-hsa-C/EBPα | CCAAGAAGUCGGUGGACAAdTdT | UUGUCCACCGACUUCUUGGdCdC | |
| si-hsa-GSK3β | GAGCAAAUCAGAGAAAUGAdTdT | UCAUUUCUCUGAUUUGCUCdCdC | |
| si-mmu-APPBP2 | GCUACAGUGGACUAGAAUAdTdT | UAUUCUAGUCCACUGUAGCdCdT | |
| NC (negative control) | UUCUCCGAACGUGUCACGUTT | ACGUGACACGUUCGGAGAATT | |
|  | | | |
| **Primers for gene or 3′ UTR or promoter cloning (restriction enzyme sites are underlined)** | | | |
| mmu-miR-128-1 | CCGCTCGAGTGCAGTCATGCAAGCAGCTA | CCGGATATCTAGTAATGAGTTTGGCATGC | |
| hsa-POU2F1 | CGGGGTACCGCCACCATGGCGGACGGAGGAGCAGC | AGTGATATCTCACTGTGCCTTGGAGGCGG | |
| hsa-GSK3β | CGGGGTACCGCCACCATGTCAGGGCGGCCCAGAAC | AGTGATATCTCAGGTGGAGTTGGAAGCTG | |
| GSK3β 3′ UTR | ATGGAATTCGCTCTTTGTTTGCCTGACCA | ATGGGGCCCCTTCTCATGCTTCAACCAGTCA | |
| APPBP2 3′ UTR | AGTGATATCGCGATCTATAGCAATTGGGAAGA | AGTTCTAGAGGTTGAAAATGTGGGCCAGA | |
| mTOR 3′ UTR | CCGGAATTCCAGATGTGCCCATCACGTTT | CCGGATATCTATGTTTAAAATTCTGATGTC | |
| p-miR-128-2 | CCGGAGCTCTGCAGAGCTACAGGTTCACA | CCGGATATCGTTCGTGCTGCTCTTTGGAT | |
| p-miR-128-1 or p-(-2/-0.06 k) | CCGCTCGAGGCTAGACTTGTCCTTTCAGCTG | CCCAAGCTTGCTACACAGAAAACAGCTAGGG | |
| p-(-1.5/-0.06 k) | CCGCTCGAGCGCACACCTGTAATACCAGC | CCCAAGCTTGCTACACAGAAAACAGCTAGGG | |
| p-(-0.8/-0.06 k) | CCGCTCGAGTTGGTGGATAGGGAGGAGGA | CCCAAGCTTGCTACACAGAAAACAGCTAGGG | |
| p-(-0.5/-0.06 k) | CGGGGTACCTCCTGATGGCCCTTAGTCAT | CCCAAGCTTGCTACACAGAAAACAGCTAGGG | |
| p-del1 | CCGCTCGAGCGCACACCTGTAATACCAGC | CCCAAGCTTGCTACACAGAAAACAGCTAGGG | |
| p-del2 | CCGCTCGAGCGCACACCTGTAATACCAGC | CCCAAGCTTGCTACACAGAAAACAGCTAGGG | |
|  |  |  | |
| **Primers for miRNA RT-qPCR** | | | |
| miR-128-RT | GTCGTATCCAGTGCAGGGTCCGAGGTATTCGCACTGGATACGACAAAGAG | | |
| U6-RT | AAAATATGGAACGCTTCACGAATTTG | | |
| miR-128-qPCR | CACGCTTCACAGTGAACCGGT | | CCAGTGCAGGGTCCGAGGTA |
| U6-qPCR | CTCGCTTCGGCAGCACATATACT | | ACGCTTCACGAATTTGCGTGTC |
|  | | | |
| **Primers for ChIP** | | | |
| C/EBPα binding site | CAGAAGAAACCCTGGCTACAC | ACACAGAGGCTTCAACAGAC | |
|  | | | |
| **Primers for qPCR** | | | |
| mmu-APPBP2 | CTAAACATGGCCAGCAAGCA | TCACGTTTGACTACACAAGCC | |
| hsa-APPBP2 | TGCACCTGTCTTCACTCCAA | AAGAAGCCAGATGTCCCACT | |
| mmu-ARPP21 | TGTCTGAGCAAGGAGGACTG | TGATCCTGGTGACTTTCCCC | |
| mmu-R3HDM1 | AAGGATCTCTGCCCACAACA | TGGTAGGTTGAGCTGCATCA | |
| hsa-C/EBPα | AACACGAAGCACGATCAGTCC | CTCATTTTGGCAAGTATCCGA | |
| mmu-C/EBPα | AGCTTACAACAGGCCAGGTTTC | CGGCTGGCGACATACAGTAC | |
| mmu-GAPDH | TGACGTGCCGCCTGGAGAAA | AGTGTAGCCCAAGATGCCCTTCAG | |
| mmu-β-actin | TGGAATCCTGTGGCATCCATGAAAC | TAAAACGCAGCTCAGTAACAGTCCG | |
| hsa-GAPDH | AGGGGCCATCCACAGTCTTC | AGAAGGCTGGGGCTCATTTG | |

**Table S3. Candidate TFs that may regulate *MIR128* transcription**

| **TF** | **Number of predicted**  **TF binding sites within the 2 kb region upstream of *MIR128*** | | **Relevance to AD** | **References** |
| --- | --- | --- | --- | --- |
|  | ***MIR128-1*** | ***MIR128-2*** |  |  |
| C/EBPα | 25 | 18 | AD risk variants and AD heritability were significantly enriched at DNA binding motifs for specific TFs including C/EBPα. | [[2](#_ENREF_2)] |
| POU2F1 (or Oct-1) | 18 | 14 | A polymorphism in POU2F1 was associated with AD | [[3](#_ENREF_3)] |
| Sp1 | 12 | 12 | Sp1 was upregulated in AD brains | [[4](#_ENREF_4)] |
| GATA-1 | 11 | 11 | GATA-1 regulated the transcription of APP and γ-secretase activating protein | [[5](#_ENREF_5), [6](#_ENREF_6)] |

TF, transcription factor. C/EBPα, CCAAT-enhanced-binding protein α. POU2F1, POU domain, class 2, transcription factor 1. Sp1, specificity protein 1. GATA-1, GATA-binding factor 1.

Transcription factor binding sites prediction:

Alibaba2.1 (<http://www.gene-regulation.com/pub/programs/alibaba2/index.html>)

**Table S4. Summary of miR-128 expression changes in AD**

| **Sample type** | **Sample size (subject distribution)** | **Post-mortem interval** | **Methods** | **Expression** | **P value** | **Disease status** | **References** |
| --- | --- | --- | --- | --- | --- | --- | --- |
| Brain-temporal neocortex (Brodmann areas 6 and 22) | 6 controls,  12 AD  (UK) | unknown | RT-qPCR | down | 0.006 | unknown | [[7](#_ENREF_7)] |
| Brain-hippocampal CA1 region | 5 fetal,  5 late adult,  5 AD  (USA) | ≤ 3 h | DNA array, Northern blot | up | 0.025 | CDR2/3 | [[8](#_ENREF_8)] |
| Brain-hippocampus | 23 controls,  41 AD  (Netherlands & UK) | unknown | nCounter miRNA assay (Nanostring) | down | 0.000000142 | Braak V/VI | [[9](#_ENREF_9)] |
| Brain-hippocampus | 11 controls,  10 AD (Braak III/IV),  10 AD (Braak VI)  (Netherlands) | 3 h to 6 h | RT-qPCR | down | 0.025 | Braak VI | [[10](#_ENREF_10)] |
| Brain-superior temporal neocortex (Brodmann area 22) | 5 controls,  5 AD  (USA) | ≤ 2 h | Sequencing | up | 0.025 | Moderate-to-late stage | [[11](#_ENREF_11)] |
| Whole blood-lymphocytes & monocytes | 37 controls,  34 AD  (Italy) | N/A | RT-qPCR | up | 0.025 | CDR2 | [[12](#_ENREF_12)] |
| Whole blood | 55 controls,  49 AD  (German) | N/A | Next-generation sequencing | up | 0.0035438 | Moderate stage | [[13](#_ENREF_13)] |
| Serum | 106 controls,  117 AD  (China) | N/A | RT-qPCR | up | < 0.01 | unknown | [[14](#_ENREF_14)] |

CDR: Clinical Dementia Rating.

**Supplementary References**

1. Su H, Yang JR, Xu T, Huang J, Xu L, Yuan Y, et al (2009) MicroRNA-101, down-regulated in hepatocellular carcinoma, promotes apoptosis and suppresses tumorigenicity. Cancer Res 69:1135-1142.

2. Tansey KE, Cameron D, Hill MJ (2018) Genetic risk for Alzheimer's disease is concentrated in specific macrophage and microglial transcriptional networks. Genome Med 10:14.

3. Taguchi K, Yamagata HD, Zhong WT, Kamino K, Akatsu H, Hata R, et al (2005) Identification of hippocampus-related candidate genes for Alzheimer's disease. Ann Neurol 57:585-588.

4. Citron BA, Dennis JS, Zeitlin RS, Echeverria V (2008) Transcription factor Sp1 dysregulation in Alzheimer's disease. J Neurosci Res 86:2499-2504.

5. Ge YW, Ghosh C, Song W, Maloney B, Lahiri DK (2004) Mechanism of promoter activity of the beta-amyloid precursor protein gene in different cell lines: identification of a specific 30 bp fragment in the proximal promoter region. J Neurochem 90:1432-1444.

6. Chu J, Wisniewski T, Pratico D (2016) GATA1-mediated transcriptional regulation of the gamma-secretase activating protein increases Abeta formation in Down syndrome. Ann Neurol 79:138-143.

7. Culpan D, Kehoe PG, Love S (2011) Tumour necrosis factor-alpha (TNF-alpha) and miRNA expression in frontal and temporal neocortex in Alzheimer's disease and the effect of TNF-alpha on miRNA expression in vitro. Int J Mol Epidemiol Genet 2:156-162.

8. Lukiw WJ (2007) Micro-RNA speciation in fetal, adult and Alzheimer's disease hippocampus. Neuroreport 18:297-300.

9. Lau P, Bossers K, Janky R, Salta E, Frigerio CS, Barbash S, et al (2013) Alteration of the microRNA network during the progression of Alzheimer's disease. EMBO Mol Med 5:1613-1634.

10. Muller M, Kuiperij HB, Claassen JA, Kusters B, Verbeek MM (2014) MicroRNAs in Alzheimer's disease: differential expression in hippocampus and cell-free cerebrospinal fluid. Neurobiol Aging 35:152-158.

11. Lukiw WJ, Surjyadipta B, Dua P, Alexandrov PN (2012) Common micro RNAs (miRNAs) target complement factor H (CFH) regulation in Alzheimer's disease (AD) and in age-related macular degeneration (AMD). Int J Biochem Mol Biol 3:105-116.

12. Tiribuzi R, Crispoltoni L, Porcellati S, Di Lullo M, Florenzano F, Pirro M, et al (2014) miR128 up-regulation correlates with impaired amyloid beta(1-42) degradation in monocytes from patients with sporadic Alzheimer's disease. Neurobiol Aging 35:345-356.

13. Keller A, Backes C, Haas J, Leidinger P, Maetzler W, Deuschle C, et al (2016) Validating Alzheimer's disease micro RNAs using next-generation sequencing. Alzheimers Dement 12:565-576.

14. Zhang M, Han W, Xu Y, Li D, Xue Q (2021) Serum miR-128 Serves as a Potential Diagnostic Biomarker for Alzheimer's Disease (vol 17, pg 269, 2021). Neuropsych Dis Treat 17:513-513.
