# Supplementary material for: MicroRNA‐128 suppresses tau phosphorylation and reduces amyloid‐beta accumulation by inhibiting the expression of GSK3β, APPBP2, and mTOR in Alzheimer's disease
Source: CNS Neurosci Ther. 2023 Mar 7;29(7):1848–64. doi: 10.1111/cns.14143 (PMC10324361; doi:10.1111/cns.14143)
Supplement: Supplementary file 2 — Figure S1: [file CNS-29-1848-s001.docx]

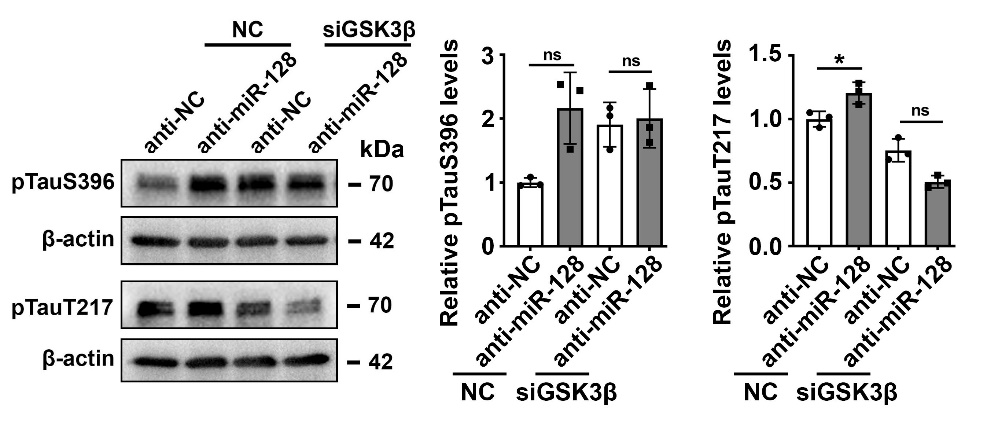


**FIGURE S1** **Knockdown of GSK3β suppresses tau phosphorylation induced by miR-128 inhibition.** 293T-Tau cells were cotransfected with anti-NC or anti-miR-128, and NC or GSK3β siRNAs (siGSK3β) for 48 hours according to the following combinations: NC/anti-NC (first bar), NC/anti-miR-128 (second bar), siGSK3β/anti-NC (third bar), or siGSK3β/anti-miR-128 (fourth bar), followed by immunoblotting analysis. Data are presented as mean ± SD. ******P* < 0.05, ns, non-significant; One-way ANOVA.

**
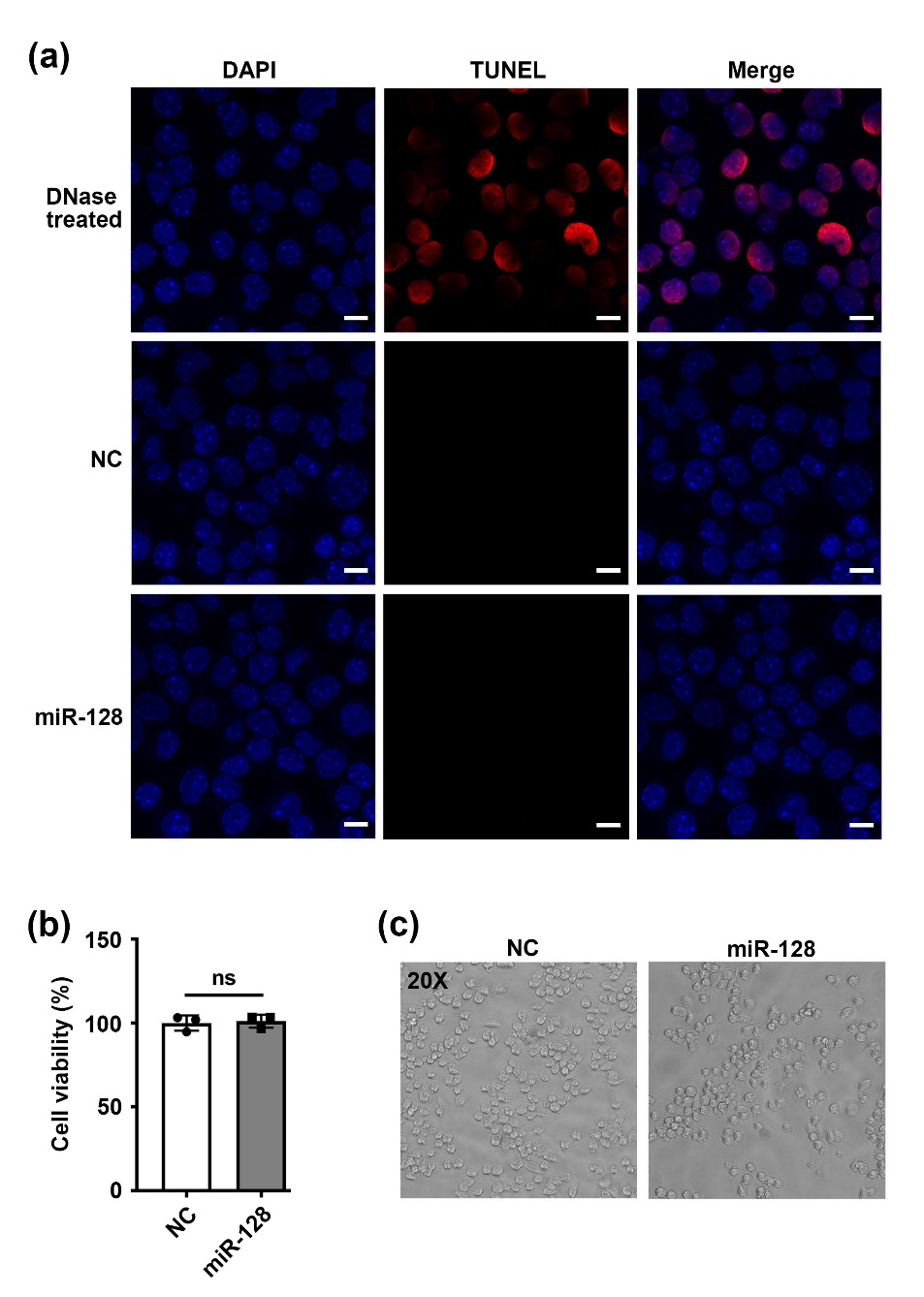
**

**FIGURE S2** **miR-128 neither induces cell apoptosis nor inhibits cell proliferation.** N2a-APPsw cells were transfected with NC or miR-128 duplexes for 24 hours, and the medium was then replaced with a fresh serum-reduced medium containing 0.2% FBS. After 24 hours incubation, cells were subjected to TUNEL assay to analyze cell apoptosis (a), XTT assay to measure cell proliferation (b), or observed under light microscopy to evaluate cell morphology (c). (a) DNase-treated cells were used as positive controls. Scale bar, 10 μm. n = 2. (b) n = 3. Data are presented as mean ± SD. ns, non-significant; Student’s *t* test for results in (b).

**
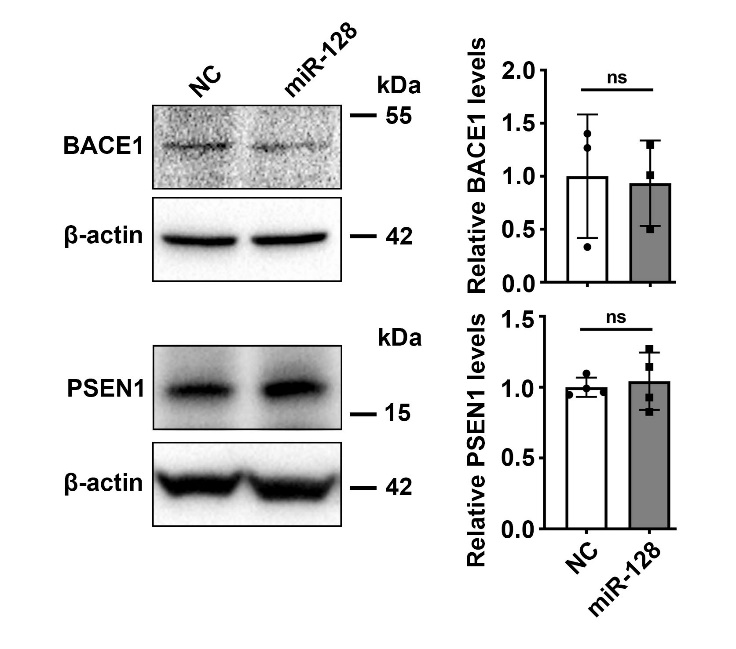
**

**FIGURE S3 Expression of BACE1 and PSEN1 are not repressed by miR-128.** N2a-APPsw cells transfected with NC or miR-128 duplexes for 48 hours were analyzed by immunoblotting. n = 3-4. Data are presented as mean ± SD. ns, non-significant; Student’s *t* test.

**
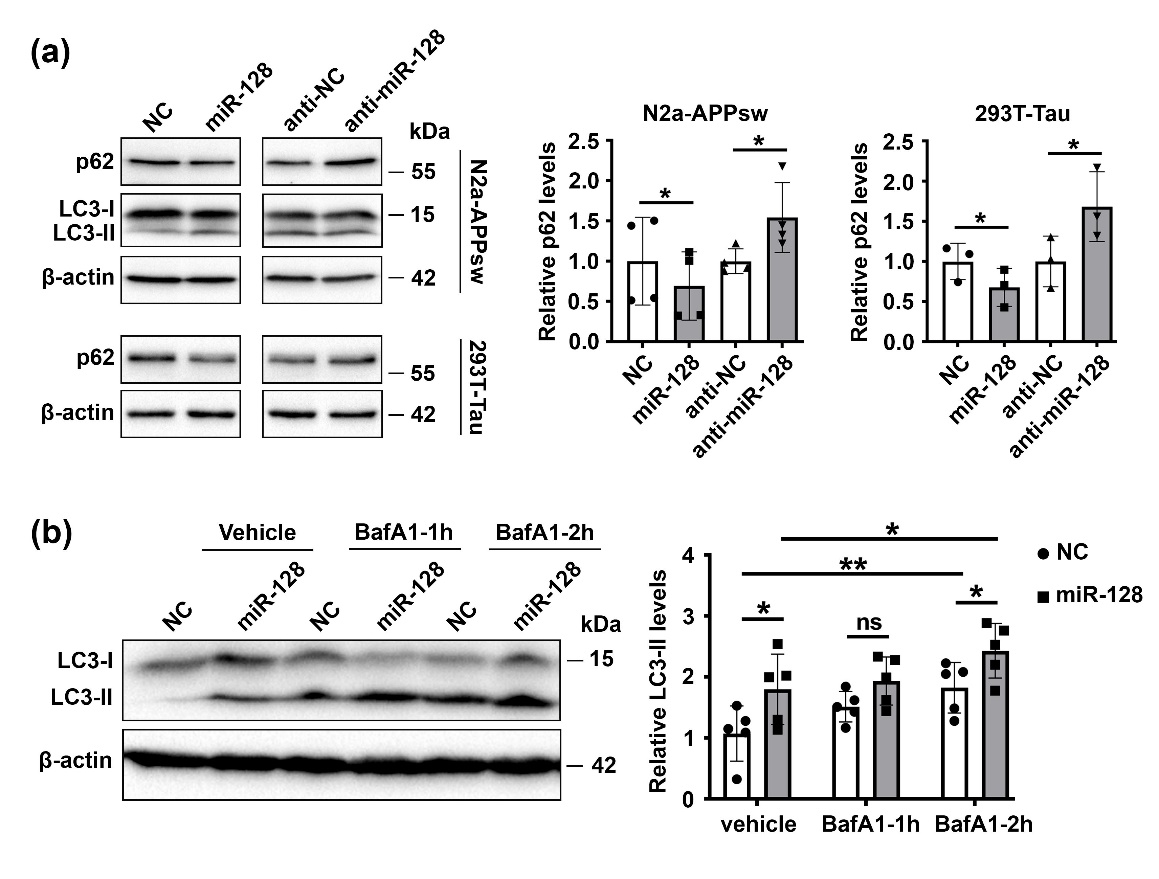
**

**FIGURE S4 Upregulation of miR-128 enhances autophagic activity.** (a) Overexpression of miR-128 downregulated p62 expression, while inhibition of miR-128 upregulated the expression. N2a-APPsw cells or 293T-Tau cells were transfected with NC/miR-128 duplex or anti-NC/anti-miR-128 for 48 hours, followed by immunoblotting analysis. (b) Bafilomycin A1 treatment or miR-128 overexpression increased LC3-II expression, and combination of the two treatments significantly enhanced the increase. N2a-APPsw cells were transfected with NC or miR-128 duplex for 48 hours and then subjected to Bafilomycin A1 (BafA1) treatment (100 nM) for 1 hour or 2 hours, followed by immunoblotting analysis. Data are presented as mean ± SD. ******P* < 0.05, ***P* < 0.01, ns, non-significant; Student’s *t* test for results in (a), Two way ANOVA for results in (b).

**
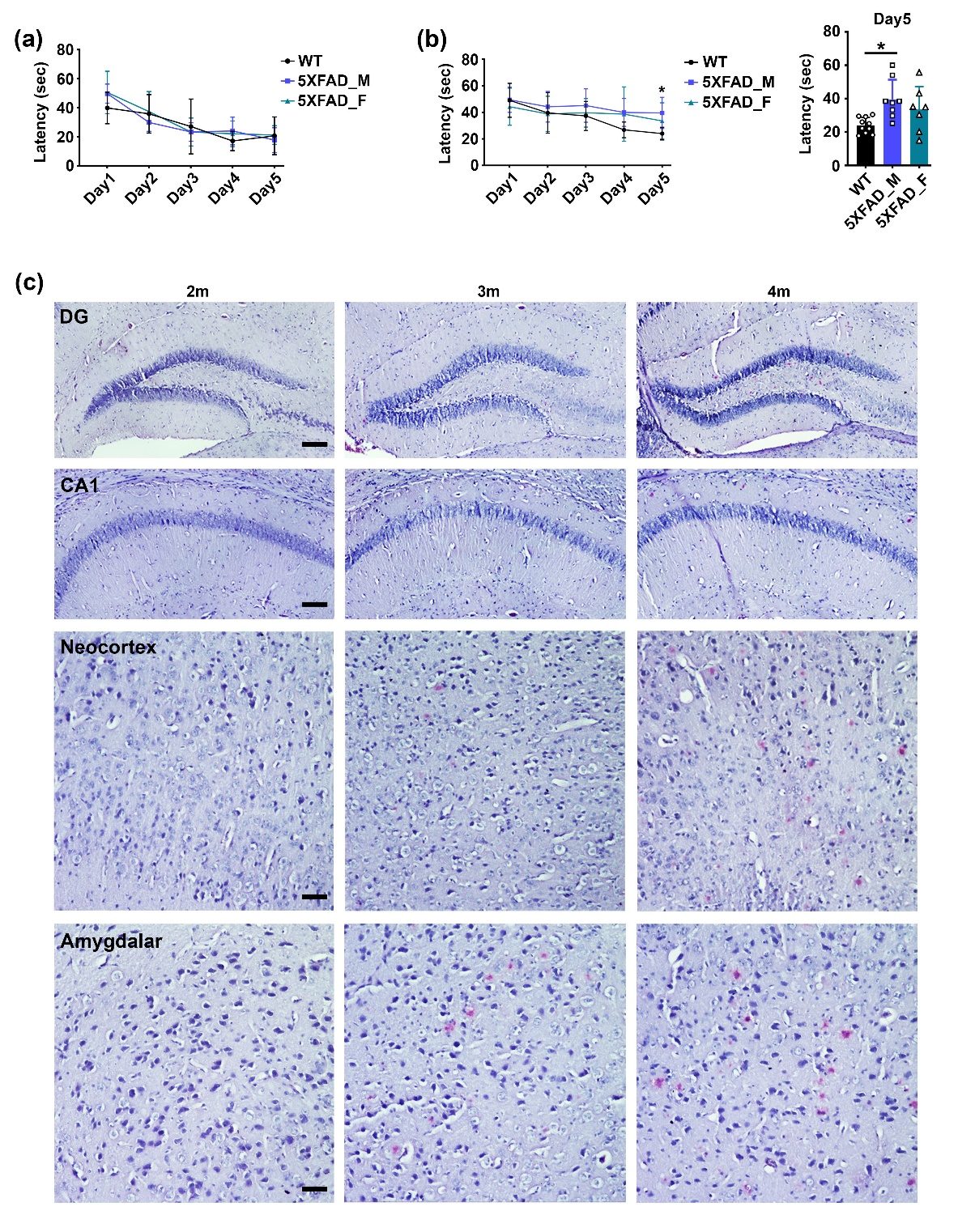
**

**FIGURE S5 Cognitive deficit and Aβ deposition in 5XFAD mice with age.** (a, b) Morris water maze test of 4-month-old mice (a) and 6-month-old mice (b). There was significant cognitive impairment in 6-month-old male 5XFAD mice (5XFAD_M) but not female 5XFAD mice (5XFAD_F) compared to age-matched wild-type mice (WT). (a) WT, n = 6; 5XFAD_M, n = 7; 5XFAD_F, n = 6. (b) WT, n = 10; 5XFAD_M, n = 8; 5XFAD_F, n = 7. (c) Immunohistochemical staining with anti-Aβ antibody in brain tissue sections of 5XFAD mice. 2m, 2-month-old 5XFAD; 3m, 3-month-old 5XFAD; 4m, 4-month-old 5XFAD. DG, dentate gyrus; CA1, cornu ammonis 1. Scale bar, 100 μm in DG and CA1, 50 μm in neocortex and amygdala. n = 3-5. Data are presented as mean ± SD. ******P* < 0.05; One-way ANOVA for results in (b).

**
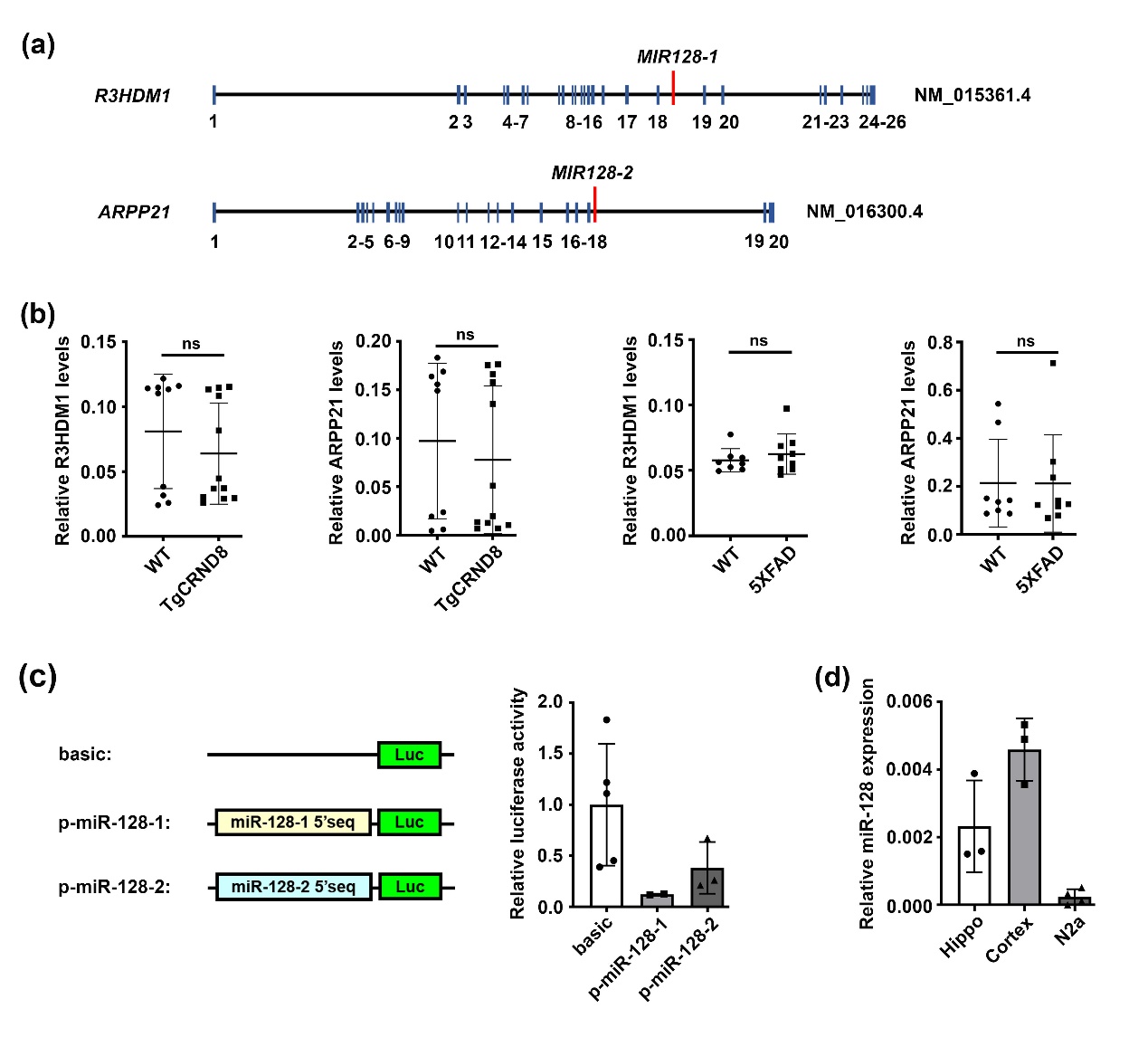
**

**FIGURE S6 Transcription of miR-128 is independent of host genes in AD mouse models.** (a) Diagram showing localization of miR-128 gene within intronic sequences of the host genes. (b) RT-qPCR results revealed that the mRNA levels of R3HDM1 and ARPP21 in the hippocampus remained unchanged between 9-month-old wild-type mice and AD transgenic mice. (c) Luciferase reporter assay showed there was no increase in luciferase activity in N2a cells transfected with miR-128 promoter reporters. (d) RT-qPCR results indicated low endogenous miR-128 levels in N2a cells. Data are presented as mean ± SD. ns, non-significant; Student’s *t* test for results in R3HDM1 levels of WT & 5XFAD from (b), Mann-Whitney test for other results from (b).

**
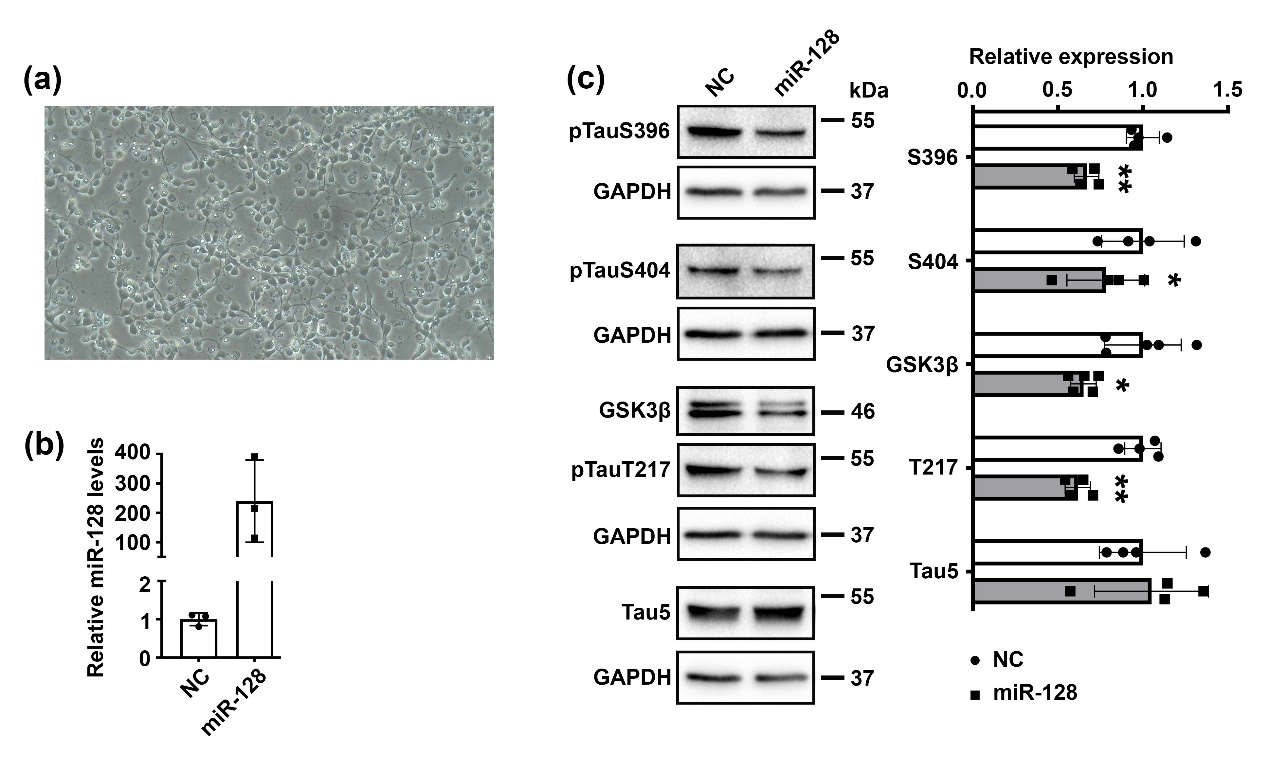
**

**FIGURE S7 Upregulation of miR-128 in primary mouse neurons inhibits tau phosphorylation and GSK3β expression.** (a) Light microscopy showing cell morphology at DIV3. (b) RT-qPCR results revealed that miR-128 expression was upregulated in cells transfected with miR-128 mimics. Mouse primary cortical neurons at DIV3 were transfected with NC or miR-128 duplex for 48 hours, followed by RT-qPCR assay. (c) miR-128 suppressed tau phosphorylation and GSK3β expression but did not affect total tau (Tau5) expression. Mouse primary cortical neurons transfected with NC or miR-128 duplex for 48 hours were analyzed by immunoblotting. GAPDH was used as a loading control. Data are presented as mean ± SD. **P* < 0.05, ***P* < 0.01; Student’s *t* test for results in (c).

**
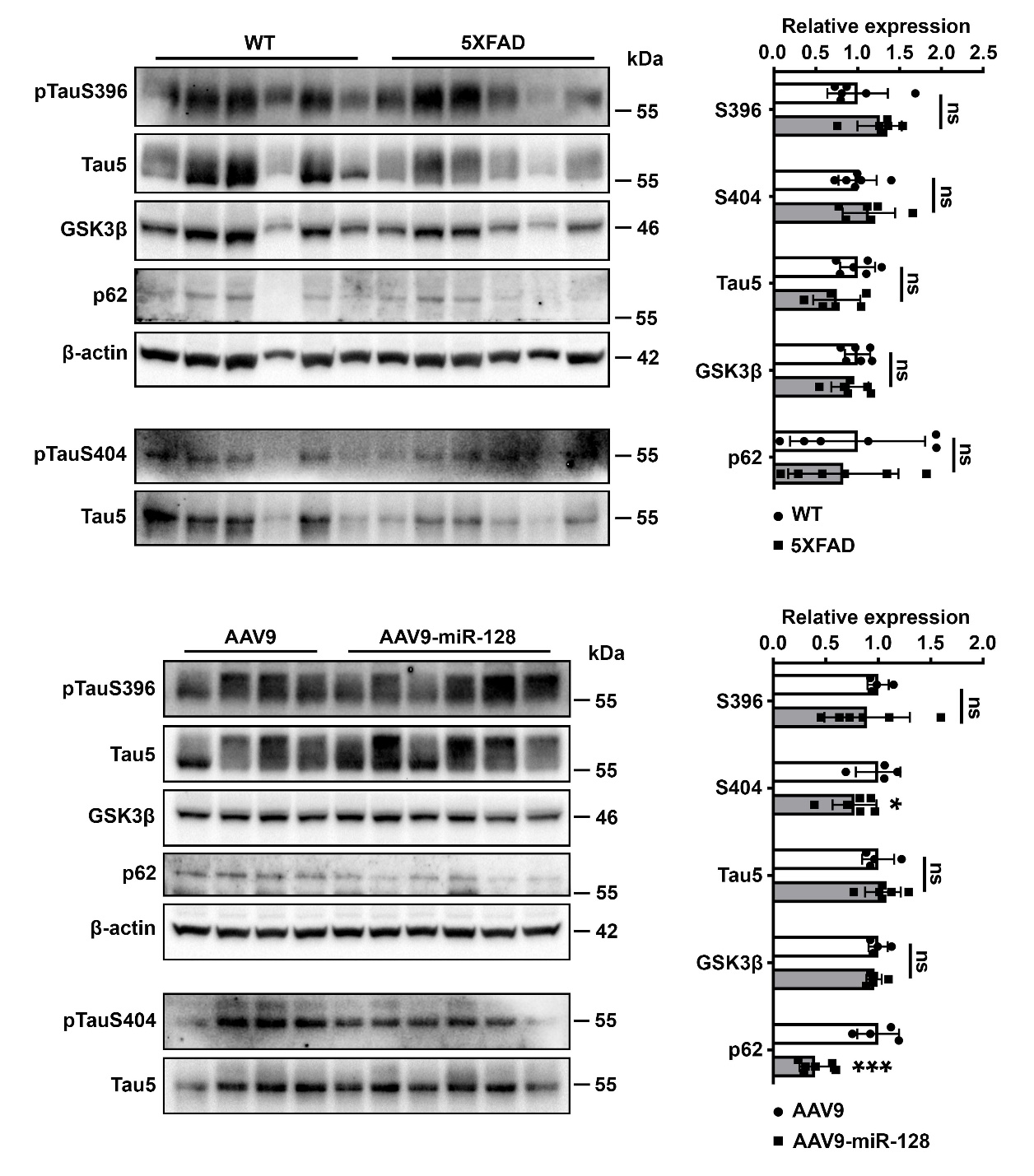
**

**FIGURE S8 *In vivo* upregulation of miR-128 does not affect GSK3β and total tau expression, but decreases phosphorylated tau and p62 expression.** The hippocampus of mice was homogenized and subjected to Western blotting. The relative expression levels of phosphorylated tau were normalized to the total tau (Tau5) and the others were normalized to β-actin. WT, wild-type mice, n = 6; 5XFAD, untreated 5XFAD mice, n = 6; AAV9, 5XFAD mice injected with empty virus, n = 4; AAV9-miR-128, 5XFAD mice injected with miR-128-expressing virus, n = 6. Data are presented as mean ± SD. ns, non-significant; Mann-Whitney test for results in pTauS396 levels of WT & 5XFAD and pTauS404 levels of AAV9 & AAV9-miR-128, Student’s *t* test for other results.

**
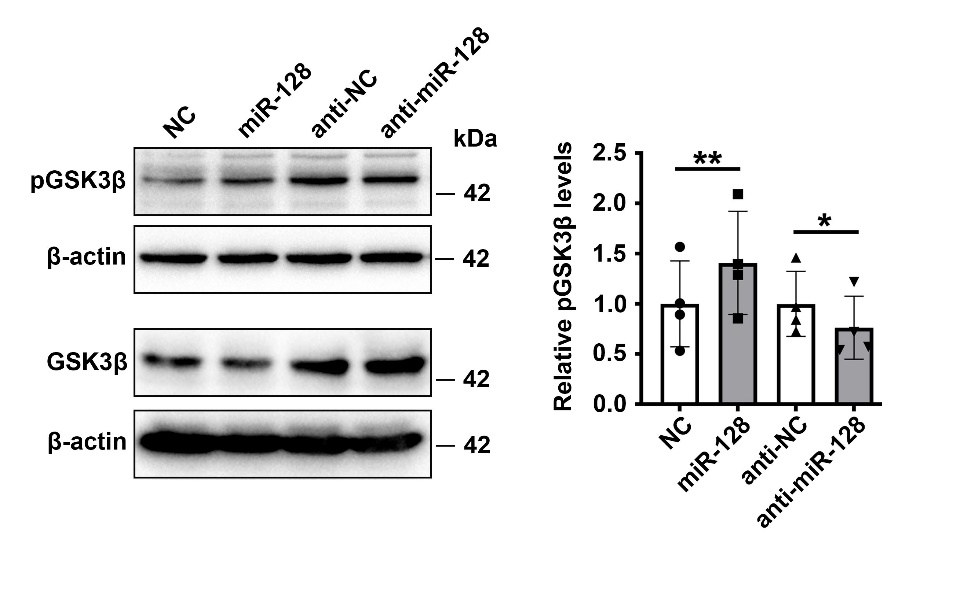
**

**FIGURE S9 Overexpression of miR-128 increases GSK3β phosphorylation.** 293T-Tau cells were transfected with NC/miR-128 duplex or anti-NC/anti-miR-128 for 48 hours, followed by immunoblotting analysis. Data are presented as mean ± SD. ******P* < 0.05, ***P* < 0.01; Student’s *t* test.

**
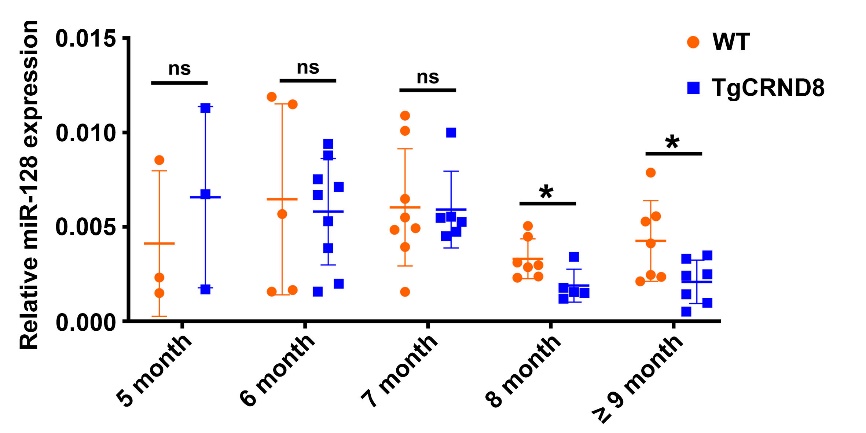
**

**FIGURE S10 Expression of miR-128 is downregulated in the hippocampus of older TgCRND8 mice.** The expression levels of mature miR-128 in the hippocampus of WT and TgCRND8 mice at different ages were analyzed using RT-qPCR. Data are presented as mean ± SD. ******P* < 0.05, ns, non-significant; Mann-Whitney test for results in 7 month and 8 month, Student’s *t* test for results in other months.
